# A microRNA Cluster Controls Fat Cell Differentiation and Adipose Tissue Expansion By Regulating SNCG

**DOI:** 10.1002/advs.202104759

**Published:** 2021-12-13

**Authors:** Ruth Rodríguez‐Barrueco, Jessica Latorre, Laura Devis‐Jáuregui, Aina Lluch, Nuria Bonifaci, Francisco J. Llobet, Mireia Olivan, Laura Coll‐Iglesias, Katja Gassner, Meredith L. Davis, José M. Moreno‐Navarrete, Anna Castells‐Nobau, Laura Plata‐Peña, Miki Dalmau‐Pastor, Marcus Höring, Gerhard Liebisch, Vesa M. Olkkonen, Maria Arnoriaga‐Rodríguez, Wifredo Ricart, José M. Fernández‐Real, José M. Silva, Francisco J. Ortega, David Llobet‐Navas

**Affiliations:** ^1^ Molecular Mechanisms and Experimental Therapy in Oncology‐Oncobell Program Bellvitge Biomedical Research Institute (IDIBELL) L'Hospitalet de Llobregat 08908 Spain; ^2^ Anatomy Unit Department of Pathology and Experimental Therapy School of Medicine University of Barcelona (UB) L'Hospitalet de Llobregat 08907 Spain; ^3^ Department of Diabetes Endocrinology, and Nutrition (UDEN) Institut d'Investigació Biomèdica de Girona (IDIBGI) Salt 17190 Spain; ^4^ Centro de Investigación Biomédica en Red de la Fisiopatología de la Obesidad y la Nutrición (CIBEROBN) Instituto de Salud Carlos III (ISCIII) Madrid 28029 Spain; ^5^ Centro de Investigación Biomédica en Red de Cáncer (CIBERONC) Instituto de Salud Carlos III, (ISCIII) Madrid 28029 Spain; ^6^ Department of Pathology Duke University School of Medicine Durham NC 27710 USA; ^7^ MIFAS by GRECMIP (Minimally Invasive Foot and Ankle Society) Merignac 33700 France; ^8^ Institute of Clinical Chemistry and Laboratory Medicine Regensburg University Hospital Regensburg 93053 Germany; ^9^ Minerva Foundation Institute for Medical Research (Biomedicum 2U) and Department of Anatomy Faculty of Medicine University of Helsinki Helsinki 00290 Finland; ^10^ Department of Pathology Icahn School of Medicine at Mount Sinai New York NY 10029 USA

**Keywords:** *γ*‐Synuclein, adipocytes, adipose tissue, miR‐424(322)/503, obesity

## Abstract

The H19X‐encoded miR‐424(322)/503 cluster regulates multiple cellular functions. Here, it is reported for the first time that it is also a critical linchpin of fat mass expansion. Deletion of this miRNA cluster in mice results in obesity, while increasing the pool of early adipocyte progenitors and hypertrophied adipocytes. Complementary loss and gain of function experiments and RNA sequencing demonstrate that miR‐424(322)/503 regulates a conserved genetic program involved in the differentiation and commitment of white adipocytes. Mechanistically, it is demonstrated that miR‐424(322)/503 targets *γ*‐Synuclein (SNCG), a factor that mediates this program rearrangement by controlling metabolic functions in fat cells, allowing adipocyte differentiation and adipose tissue enlargement. Accordingly, diminished miR‐424(322) in mice and obese humans co‐segregate with increased SNCG in fat and peripheral blood as mutually exclusive features of obesity, being normalized upon weight loss. The data unveil a previously unknown regulatory mechanism of fat mass expansion tightly controlled by the miR‐424(322)/503 through SNCG.

## Graphical abstract

Absence of H19X‐encoded miR‐424(322)/503 in mice results in altered progenitor/adipose stem cell homeostasis, adipose tissue expansion, and its expression is reduced in obese subjects. Mechanistically, epigenetically‐controlled miR‐424(322)/503 modulates adipocyte differentiation by regulating *γ*‐Synuclein (SNCG). Expression levels of miR‐424(322)/503 and tissular/soluble SNCG co‐segregate with obesity in humans and can be modulated by weight loss surgically induced by gastric bypass.

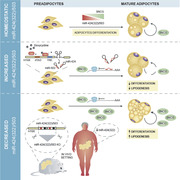

## 1 Introduction

Whilst factors determining adipose tissue expansion (hyperplasia) are not fully understood, increased lipid storage in mature adipocytes (hypertrophy) is thought to be of the utmost importance for obesity‐related diseases.^[^ ^1^, ^2^ ^]^ Absolute fat cell production and adipocyte turnover show that obese individuals appear to have a greater number of adipocytes than lean individuals,^[^ ^3^, ^4^ ^]^ being this difference set before adulthood.^[^ ^4^ ^]^ As the turnover of new‐born adipocytes added each year is almost the same for both groups, loss of fat cells seems to be largely compensated by the production of new adipocytes, which is twice as high in obese as compared with lean individuals.^[^ ^5^ ^]^ In line with this, lipid removal rate in fat depots decreases with age. Failure to adjust the rate of fatty acid biosynthesis and uptake results in weight gain, whereas substantial surgery‐induced weight loss is mainly driven by significant changes in lipid storage.^[^ ^6^, ^7^ ^]^ In such scenarios, the pivotal relevance of adipocytes and the number of committed fat precursor cells,^[^ ^8^ ^]^ as well as the ability to maintain functionality and properly differentiate into adipocytes (adipogenesis), appear to influence physiology to the extent of altering the systemic metabolic state.^[^ ^9^, ^10^ ^]^

During adipogenesis, precursor mesenchymal stem cells and preadipocytes found within white adipose tissue (WAT) differentiate into cells able to synthesize and store massive amounts of triglycerides. In fact, the main feature of adipocytes is the ability to package the energy surplus into large lipid droplets, while awaiting concrete systemic signals to mobilize nutrients as needed by the organism.^[^ ^11^ ^]^ Exposure to calorie‐dense diets, accompanied by infrequent or poor physical activity, results in an unremitting caloric surplus that characterizes the Western world. Enhanced adipogenesis and adipose tissue expansion not only distribute energy among newly formed adipocytes,^[^ ^9^ ^]^ but also increase the number of hypertrophied adipocytes. These large adipocytes may release pro‐inflammatory cytokines related to the hypoxic stress,^[^ ^12^ ^]^ followed by recruitment and activation of immune cells.^[^ ^13^ ^]^ The adipogenic niche can also compromise tissue adipogenic capability to a point at which adipocytes transdifferentiate back into mesenchymal precursor cells.^[^ ^14^ ^]^ Such plasticity is exemplified in pregnancy, when adipocytes located at the stromal compartment of mammary glands gradually disappear in response to proliferation of the ductal epithelium and formation of milk‐secreting alveoli during lactation.^[^ ^15^, ^16^ ^]^

In recent years, the mechanistic insights on the post‐transcriptional regulation exercised by the cluster miR‐424(322)/503 have increased exponentially, with contexts spanning from cellular differentiation to proliferation and cancer.^[^ ^17^ ^]^ Our laboratory was the first to generate a miR‐424(322)/503 knockout mouse model, which significantly contributed to the functional characterization of this cluster in the context of mammary gland physiology, as a regulator of post‐lactating mammary gland involution, and as a tumor suppressor.^[^ ^18^, ^19^ ^]^ Here, we present in vivo, in vitro, and clinical and functional data to support novel evidences of a previously unknown biological function of the cluster miR‐424(322)/503. This study primarily stems from observations of significant enlargement of fat depots in the miR‐424(322)/503 knockout mouse model. We demonstrate that the miR‐424(322)/503‐null mice display increased adiposity as a result of a rapid and transient differentiation of new adipocytes found within adipose tissue, coupled to enhanced fatty acid anabolism and increased energy storage in adipose cells. Importantly, we provide evidences that this is consistent with the patterns of miRNA and target gene expression observed in humans. At the molecular level, we used complementary cell models, and human and mouse samples to identify and validate the direct impact of miR‐424(322)/503 on the expression of *γ*‐Synuclein (SNCG), a target gene involved in mediating metabolic functions in adipocytes.^[^ ^20^ ^]^ Notably, SNCG supports adipocyte differentiation and adipose tissue expansion, the function of which is particularly important in conditions of nutrient excess.^[^ ^21^ ^]^ These findings demonstrate a novel mechanism by which homeostatic miRNAs found in adipose tissue may serve as a key rheostat, modulating impaired adipogenesis and fat mass expansion through the control of SNCG.

## 2 Results

### 2.1 The miR‐424(322)/503‐Deficient Mice Display Adipose Tissue Enlargement

**Figure 1:**
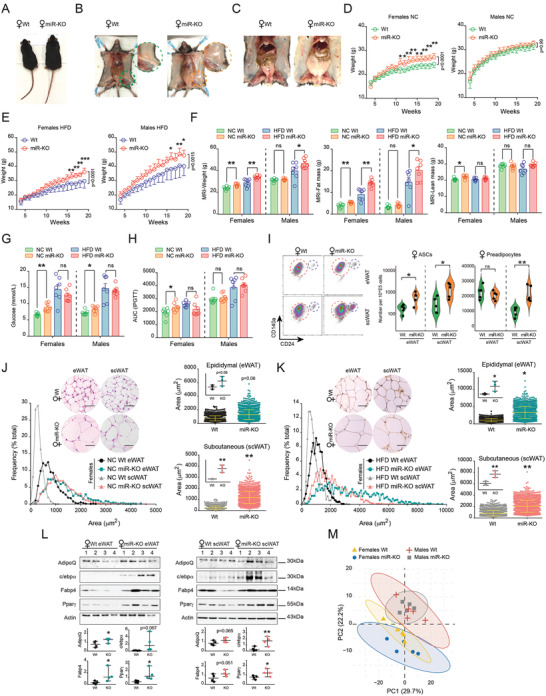
The miR‐424(322)/503 deficient mouse exhibits obesity features. A) Representative wild‐type (Wt) and miR‐424(322)/503 knockout (miR‐KO) 3‐month old female specimens on normal chow (NC). B,C) White adipose tissue enlargement was evident by means of Wt and miR‐KO female mice dissections. Growth curve of Wt and miR‐KO female and male littermates under D) NC and E) a high‐fat diet (HFD) (*n* = 7/genotype/sex). Statistical significance was assessed by adjusted ANOVA after applying Šidák's corrections to repeated measures. Family‐wise significance and confidence level were set at 0.05 (95% confidence interval). Šidák's multiple comparison test adjusted *P*‐value for each time point and *P*‐value summary for the interaction time plus treatment are provided. F) Determination of body weight, fat, and lean mass content by Magnetic Resonance Imaging (MRI). G) Glucose levels in blood after fasting, and H) quantification of the area under the curve (AUC) from intraperitoneal glucose tolerance tests (IPGTT) performed in 5 month‐old female and male Wt and miR‐KO mice (see also Figure S1E, Supporting Information) (*n* = 7/genotype/sex). I) Determination of adipose‐derived stem cells (ASCs) and pre‐adipocytes in Wt and miR‐KO female mice. Violin plots at the right show the number of ASCs and preadipocytes per 10^E5 cells sorted (*n* = 5/genotype/sex). Statistical significance was assessed by the Mann‐Whitney test. J,K) Representative images (Scale bars, 50 µm) of hematoxylin/eosin‐stained histological sections of epididymal (eWAT) and subcutaneous (scWAT) fat deposits of 5 month‐old Wt and miR‐KO female mice maintained on NC and HFD, respectively. Dot plots representing area of adipocytes and Gaussian fit histograms depicting adipocyte area distribution are provided. 500 adipocytes were measured per mouse (*n* = 3/genotype/sex, thus 1500 adipocytes/genotype/sex). L) Western blots show protein levels of adipogenic markers in fat deposits of Wt and miR‐KO female mice under NC (*n* = 4/genotype). Statistical significance between 2 groups (i.e., miR‐KO versus Wt male or female mice) was determined by Student's *t*‐test. *p < 0.05; **p < 0.01; ***p < 0.001. NS, not significant. M) Lipidomics of the scWAT of miR‐KO and Wt male and female mice (*n* = 5 per group) summarized a total of 187 lipid species. Principal component analysis (PCA) shows clear differences between males and females, and points out slightly variations between miR‐KO and Wt females (see also in Figure S2D,E, Supporting Information).

We have previously reported the generation, from a mixed C57BL/6xBALBc background, of a miR‐424(322)/503 knockout transgenic mouse model (hereafter referred to as miR‐KO mice) to study the role of this polycistronic miRNA cluster during mammary gland involution.^[^ ^18^ ^]^ Through direct observation, we noticed that adult miR‐KO mice become larger than wild type (Wt) counterparts by the accumulation of white adipose tissue (WAT). Importantly, this was consistent with our reported expression analysis across mouse tissues, which demonstrates a significant expression level of the miR‐424(322) in WAT.^[^ ^18^ ^]^ In order to characterize this phenotype further, we resolved to backcross our mixed C57BL/6xBALBc miR‐KO mice with Wt C57BL/6 mice to obtain miR‐KO mice on a >99.5% clean C57BL/6 background. This model is known to represent a more permissive genetic strain to the development of diet‐induced obesity and diabetes.^[^ ^22^ ^]^ Phenotypically, miR‐KO pups were identical to Wt in appearance, size, and weight. However, at a more advanced age (3 months), miR‐KO females showed higher body size (**Figure** **1**A) due to an apparent increased accumulation of WAT, affecting depots of both subcutaneous (beneath the skin) and omental (the bulk of mesenteric, perigonadal and perirenal adipose mass demarcated by the peritoneum) fat (Figure 1B,C and Figure S1A, Supporting Information). This observation led us to investigate whether the cluster miR‐424(322)/503 could play a role in the development of obesity. To address this, 7 week‐old male and female miR‐KO and Wt mice were maintained with a normal chow (NC) or challenged with a high‐fat diet (HFD), and monitored for a period of 14 weeks. Although Wt and miR‐KO males showed no statistically significant differences in weight gain under NC, miR‐KO females displayed a progressive and significant increase in body weight over time (Figure 1D). Remarkably, both HFD‐fed male and female miR‐KO mice gained substantially more weight than their Wt counterparts (Figure 1E). Quantitative magnetic resonance imaging (MRI) analysis of body composition confirmed that the increase in body size and whole body weight in adult miR‐KO mice fed NC (only females) and HFD (both males and females) was consistent with a significant increase in fat mass (Figure 1F). This increase was supported by dual‐energy X‐ray absorptiometry (DEXA) performed on Wt and miR‐KO mice given NC, which also indicated no differences in bone mineral density and content influencing the increased body mass in either males or females (Figure S1B, Supporting Information), nor significant variations in body length (Figure S1C, Supporting Information).

To gain insight into the metabolic consequences of miR‐424(322)/503 depletion, we measured the concentration of circulating glucose (Figure 1G), cholesterol, triglycerides, and free fatty acids (FFA) at fasting (Figure S1D, Supporting Information). Additionally, we performed intraperitoneal glucose tolerance tests (IPGTT) in miR‐KO and Wt adult mice following either NC or HFD (Figure 1H). Notably, miR‐KO mice fed NC (but not the HFD) exhibited increased fasting glucose levels in blood (both males and females), and female animals showed traits of insulin resistance (Figure S1E, Supporting Information). Such differences in the metabolic state could stem from significant variations in fat cell distribution and adipose function, including potential to undergo rapid lipid turnover, differentiation of adipocyte precursor cells, and expansion of fat depots via adipogenesis.^[^ ^1^, ^8^, ^23^ ^]^ Therefore, we resolved to analyze the content of functional immature adipocyte progenitors in Wt and miR‐KO mice. To achieve this, we enzymatically digested isolated adipose tissue to generate a single cell suspension enriched for immune, endothelial and mesenchymal cells known as the stromal vascular fraction (SVF).^[^ ^24^ ^]^ We then analyzed the SVF of miR‐KO and Wt adult females by flow cytometry to assess changes in the population of resident Lin^−^/CD34^+^/Sca‐1^+^/CD140a(PDGFRa)^+^/CD24^+^ early adipocyte progenitor cells/ASCs (adipose stem cells) and CD24^−^ preadipocytes^[^ ^25^ ^]^ (Figure S1F, Supporting Information). Interestingly, we found that the fraction of preadipocytes and ASCs were significantly higher in subcutaneous (scWAT) and epididymal (eWAT) adipose deposits from miR‐KO females, compared to their age, sex, and diet‐paired Wt counterparts (Figure 1I). In line with this, expression of adipocyte commitment/preadipocyte marker genes such as *Zfp423* ^[^ ^26^ ^]^ and *Klf14* ^[^ ^27^, ^28^ ^]^ was higher in the miR‐KO mouse (Figure S1G, Supporting Information), and adipocyte‐numbers (estimated as volume of fat deposit/average adipocyte's volume) (Figure S2H, Supporting Information), coupled to site‐specific phosphorylation of core histone H3 at serine 10 (a marker to mitosis and cell proliferation^[^ ^29^, ^30^ ^]^) (Figure S2I, Supporting Information), depicted a modification of hyperplasia and enhanced commitment to the adipocyte lineage at least in female mice lacking the miR‐424(322)/503 cluster.

On the basis of these lines of evidence, we hypothesized that the increased ability of the miR‐KO mice to accumulate fat mass was due not only to changes in fat cell numbers but also to enabled adipogenesis and increased fat cell size, resulting in adipocyte hypertrophy. To test this possibility, we calculated the adipocyte area and distribution from hematoxylin/eosin‐stained histological sections using scWAT and eWAT fat deposits from miR‐KO and Wt males and females maintained on NC or HFD. This morphometric analysis showed that adipocytes in miR‐KO female mice dramatically increased in size as compared to those of Wt mice under the same dietary conditions (Figure 1J,K). We also observed notably milder differences in male mice, with only scWAT showing statistically significant differences under HFD conditions (Figure S2A,B, Supporting Information). To better characterize the increase in the putative adipose cell population, we used real time‐PCR and western blot to examine the expression of adipocyte markers. Accordingly, there was a significant increase in the expression of mRNA transcripts (e.g., *AdipoQ*, *Glut4*, *Fasn*) and protein levels related to the adipogenic program in the miR‐KO mice (Figure 1L and Figure S2C, Supporting Information). Noteworthy, lipidomics data from scWAT of miR‐KO female mice under NC showed a lipid pattern distinct from their sex and age‐matched Wt counterparts (Figure 1M), namely composition changes in short‐chain triglycerides (e.g., TG 45:1, TG 45:2), lysophosphatidylcholines (LPC 18:1, LPC 18:2, LPC 20:4), phosphatidylethanolamines (PE 36:3, PE 36:4, PE 38:4), and PE based plasmalogens (PEP 18:1/20:4, PEP 18:0/20:4), reflecting variations related to large adipocytes and the bulk lipid metabolism of adipose tissue (Figure S2D,E, Supporting Information). While unsaturated TG may stand for the glycerophospholipid content and the overrepresentation of adipocytes and early committed adipocyte precursor cells, which may play a causal role in the development of an obese yet metabolically favorable adipose tissue in conditions of HFD,^[^ ^31^, ^32^ ^]^ changes affecting LPC and PE lipid species could be related to the linoleic and/or arachidonic acid metabolism, depicting an increased capacity to accommodate high amounts of lipids in miR‐KO females, thus protecting against metabolic disturbances derived from the consumption of HFD‐derived lipids. Notwithstanding this, enhanced adipocyte growth in females lacking the cluster miR‐424(322)/503 and maintained under a normocaloric dietary regime may have a negative impact on glucose homeostasis. Altogether, these insights highlighted an apparent increase in *de novo* adipogenesis affecting the miR‐KO mouse and indicated that, in addition (or concomitantly) to its physiological effects on weight, fat mass expansion, and energy homeostasis, the cluster miR‐424(322)/503 may regulate fat cell distribution in adipose tissue.

### 2.2 The Cluster miR‐424(322)/503 Is Transcriptionally Repressed during Adipogenesis

**Figure 2:**
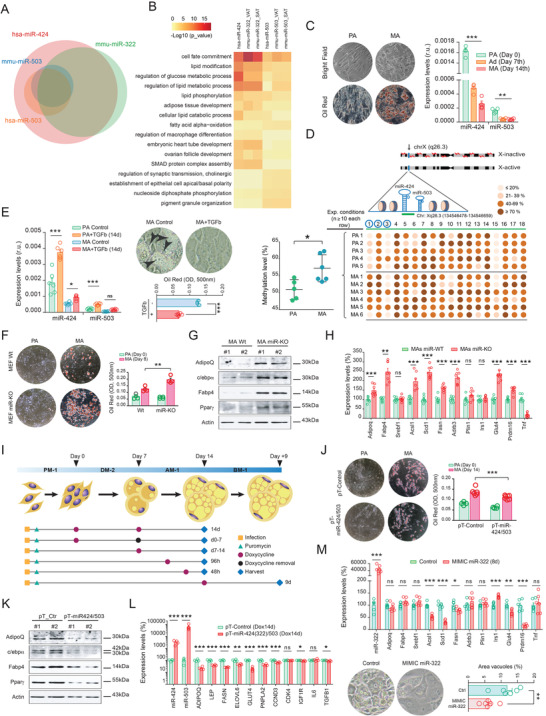
The cluster miR‐424(322)/503 reduces adipogenesis. A) Human miR‐424‐5p and miR‐503‐5p share common target genes with mouse miR‐322‐5p and miR‐503‐5p. B) Heatmap illustrating representative enriched pathways from pre‐selected human and mouse miRNA cluster targets based on AT RNA sequencing. C) Left, bright field, and oil‐red representative images of in vitro differentiation of human adipocytes. Right, expression of miR‐424(322) and miR‐503 in differentiating human preadipocytes (PA) at day 7 (Ad) and 14 (MA) after inducing adipogenesis, as explained in the Experimental Section of this article. D) Bisulfite sequencing analysis of 18 CpG in human preadipocytes and mature adipocytes. Total CpGs analyzed >2000. Upper figure illustrates the genomic location of the miR‐424(322)/503 cluster. Green bar under miR‐424 and miR‐503 represents the genomic region analyzed that contains the 18 sequential CpG islands, three of them located within the pre‐miR‐424 (CpG 1 to 3, circled in blue). Middle graph contains all biological replicates (rows). Each row/replicate encompasses the 18 CpG dinucleotides and represents the average of ≥10 sequenced alleles (see in Figure S3C, Supporting Information). Percentage of methylation for each CpG is color‐coded. Left scatter plot graph shows the percentage of methylation of each replicate (average of 18 CpG) across PA (*n* = 5) and MA (*n* = 6). E) Human preadipocytes and mature adipocytes challenged with 10 ng mL^−1^ of recombinant transforming growth factor beta (TGF*β*) for 14 days displayed enhanced expressions of miR‐424(322) and miR‐503, and impaired differentiation. Arrowheads indicate lipid droplets stained with Oil Red O (*n* = 6 biological replicates/group). F) Mouse embryonic fibroblasts (MEF) from miR‐KO mice had increased adipocyte formation (Oil Red O staining) when cultured in an adipogenic medium. G) Analysis of markers of adipogenesis (i.e., Pparg, Cebpa, Fabp4, and adiponectin) by western blot in Wt and miR‐KO adipocytes. H) Expression of a battery of genes related to the adipogenic program in mouse adipocytes derived from miR‐KO (red) and Wt (green) MEF, the latter taken as reference (100%) (see also Figure S4B, Supporting Information). I) Different stages of the differentiation of human preadipocytes were challenged by our transient doxycycline (Dox)‐inducible pTRIPz lentiviral system (pT‐miR‐424/503). PM‐1, DM‐2, AM‐1, and BM‐1 stand for preadipocyte, adipocyte differentiation, adipocyte maintenance, and adipocyte basal media, respectively. J,K) 14 day‐lasting Dox‐treated pT‐miR424/503 human adipocytes (*n* = 6) exhibit impaired adipogenesis when compared to control cells (pT‐Control, *n* = 4), as revealed by Oil Red O staining and protein levels of markers of adipogenesis (*n* = 2/group), and L) gene expression levels (*n* = 4/group), respectively (see also Figure S4C–G, Supporting Information). Data are presented as mean ± S.E.M. Statistical significance was determined by paired Student's *t*‐test. *p < 0.05; **p < 0.01; ***p < 0.001. NS, not significant.

Although conserved miRNAs show high degrees of complementarity between evolutionarily distant species,^[^ ^33^ ^]^ the 3'‐UTR miRNA binding sites may display great variations between vertebrates.^[^ ^34^ ^]^ This may compromise the cross‐species translational utility of observations made in animal models. Based on this consideration, and before proceeding with further experimental assessments, we evaluated i) the human‐mouse cross‐species conservation of target genes for the miR‐424(322) and miR‐503; and ii) given its relevant expression levels in WAT, the potential functional role for this miRNA cluster in fat depots. To this end, we first used computational predictions from the Targetscan algorithm (http://www.targetscan.org), which has one of the highest specificities in preselecting putative target genes.^[^ ^35^ ^]^ Of note, the miR‐424(322) and miR‐503 synteny is highly conserved in mammals. Both paralogs belong to the same family of miRNAs and, therefore, share a significant amount of target genes.^[^ ^36^ ^]^ In line with this, our cross‐species data analysis indicated that ≈90% of the predicted mouse targets for miR‐424(322) and miR‐503 are included within the list of their human ortholog's targets (**Figure** **2**A and Figure S3A, Supporting Information). To explore the potential conserved biological function of this miRNA cluster in the adipose tissue, we then generated a curated list of miR‐424(322)/503 target genes in mouse and human WAT using Targetscan‐inferred genes and by individually analyzing their relative expression by RNA sequencing.^[^ ^37^, ^38^ ^]^ Our pathway enrichment analysis suggested that the miR‐424(322)/503 may be involved in key human/mouse cellular processes in WAT (e.g., lipid and glucose metabolism, and adipose tissue development) (Figure 2B), outlining a potential miR‐424(322)/503 conserved function in the adipose tissue across mammals.

As the expansion of fat mass in adult mice was consistent with significant variations in fat cell size and adipocyte progenitors, we sought to study the expression patterns of miR‐424(322) and miR‐503 in primary preadipocytes representing the adherent cells of SVF isolated from human fat samples. These cells can differentiate into adipocytes when plated in culture and stimulated with adipogenic conditions.^[^ ^39^ ^]^ Importantly, we found that the expression of these miRNAs plummeted during adipocyte differentiation (Figure 2C and Figure S3B, Supporting Information). The miR‐424(322)/503 cluster is located within a CpG island in the long arm of the X‐chromosome (Xq26.3). Since the expression of the miR‐424(322)/503 cluster^[^ ^40^, ^41^ ^]^ can be epigenetically regulated by DNA methylation, we reasoned that similar repression mechanisms may explain the decrease in miR‐424(322) and miR‐503 observed during adipogenesis. To determine this, we performed bisulfite‐sequencing analysis to investigate the DNA methylation levels of the miRNA locus (Chr: Xq26.3 (134546478‐134546659), negative strand) in human preadipocytes before and after the induction of adipogenesis. Interestingly, analysis of a region spanning 18 CpG dinucleotides demonstrated that the miR‐424(322)/503 locus is highly methylated in mature adipocytes (MA) compared to preadipocytes (PA), which may explain the observed reduction in miR‐424(322) and miR‐503 expression during adipogenic commitment (Figure 2D and Figure S3C, Supporting Information). It should be noted that methylated residues located within the miR‐424(322)/503 loci overlap transcription factor binding sites sensitive to DNA methylation and likely related to the adipogenesis program (Figure S3D, Supporting Information). Next, we questioned whether stimuli that may compromise adipocyte differentiation might also lead to reciprocal changes in the expression of our miRNA candidates. For this purpose, we explored the expression levels of miR‐424(322) and miR‐503 in primary preadipocytes challenged with transforming growth factor beta (TGF*β*), a potent inhibitor of cell proliferation that blocks adipocyte development^[^ ^42^ ^]^ and acts as an inducer of the miR‐424(322)/503 cluster in other cells.^[^ ^43^ ^]^ As expected, treatment of human preadipocytes with TGF*β* resulted in a steep increase in miR‐424(322) and miR‐503 expression levels and blunted preadipocyte differentiation into mature lipid‐containing adipocytes. Importantly, the transcriptional control by TGF*β* diminished in MA (Figure 2E). This supports previous observations reporting a progressive desensitization during adipogenesis due to decreased TGF*β*‐receptor expression and availability.^[^ ^44^ ^]^ Taken together, our results highlight the potential relevance of the cluster miR‐424(322)/503 as a key rheostat in the control of molecular events leading to fatty acid biosynthesis (lipogenesis) and fat cell differentiation (adipogenesis), thus the cluster may be critical to adipose tissue development and fat mass expansion.

### 2.3 The Cluster miR‐424(322)/503 Tackles Adipogenesis

To examine the role of the cluster miR‐424(322)/503 in adipocyte differentiation, we took advantage of complementary gain and loss‐of‐function cellular models: the adipogenic induction of mouse embryonic fibroblasts (MEFs) isolated from Wt and miR‐KO mice, and human primary preadipocytes carrying a doxycycline (Dox)‐inducible pTRIPz lentiviral system (Figure S4A, Supporting Information).^[^ ^18^ ^]^ Notably, miR‐KO MEFs showed enhanced adipogenic differentiation when compared to Wt controls, as visualized by an increase in Oil Red O staining (Figure 2F). Accordingly, MA derived from miR‐KO MEFs also displayed enhanced protein levels of adipogenesis markers (Figure 2G), and increased transcriptional expression of genes related to adipocyte performance (e.g., *AdipoQ*, *Fabp4*, *Glut4*) and fatty acid metabolism (*Acsl1*, *Scd1*, *Fasn*), both under differentiating (Figure 2H) and basal cell culture conditions (Figure S4B, Supporting Information). It should also be noted that miR‐KO cells grown under non‐differentiating culture conditions did not show apparent lipid droplets in the cytoplasm (Figure 2F), suggesting that additional molecular mechanisms must be triggered to achieve adipocyte differentiation. Notwithstanding this, these results in vitro were consistent with the overall increase in committed adipocytes found within the SVF, and the adipogenic potential observed in fat samples from the miR‐KO female mice (Figure 1I–L). Current results also indicated a preference toward the adipogenic phenotype in fat cells lacking the cluster miR‐424(322)/503.

Adipogenesis can be broadly divided into two stages: i) commitment of adipocyte precursor cells, which can be induced by treating early adipocyte progenitors with an adipogenic cocktail (day 0 to 7th), and ii) terminal adipocyte differentiation (day 7 to 14th), which is dependent on insulin treatment.^[^ ^45^ ^]^ To survey the functional contribution of the cluster miR‐424(322)/503 during adipogenesis, we also used a doxycycline (dox)‐inducible pTRIPz lentiviral system to up‐regulate the levels of miR‐424(322) and miR‐503 in primary human preadipocytes, while progressing toward lipid‐filled adipocytes. This system allowed us to control the timing of miR‐424(322)/503 induction at different stages over the adipogenic course (Figure 2I and Figure S4C–G, Supporting Information). These different stages consisted of the following: i) induction over the entire assay period (14 days) in adipocyte precursor cells cultured in preadipocyte media without additional hormones (Figure S4C, Supporting Information) and in differentiating adipocytes (Figure 2J–L); ii) during the first (0–7 days) or the last (7–14 days) days of early/terminal differentiation (Figure S4D,E, Supporting Information); and iii) during the last 96/48 h of terminal differentiation (Figure S4F,G, Supporting Information). Noteworthy, transient up‐regulation of our miRNA candidates at any stage of adipocyte differentiation clearly attenuated the prevalence of adipogenic markers, as assessed by the expression of *ADIPOQ*, *LEP*, and *FASN*, among others. Next, we conducted experiments of mmu‐miR‐322 gain‐of function in 3T3‐L1 cells, and evaluated the loss of hsa‐miR‐424 in human telomerase reverse transcriptase (hTERT) immortalized human adipose‐derived mesenchymal stem cells (ASC52telo) when differentiating into white adipocytes.^[^ ^46^ ^]^ To do so, we challenged mouse adipocytes with a synthetic miRIDIAN‐Mimic of mmu‐miR‐322‐5p both during hormone‐induced adipogenesis (Figure 2M) and in a treatment of 96 h (Figure S4H, Supporting Information), and ASC52telo cells (sustained treatment lasting the adipogenic course) with a synthetic hairpin inhibitor against human hsa‐miR‐424‐5p (Figure S4I, Supporting Information). This provided substantial endorsement of the relevance of the miR‐424(322) as a critical linchpin of the adipogenic course and adipocyte performance, as proved that adipocyte precursor cells lacking the miR‐424(322) are more prone to differentiate into adipocytes, and that adipocyte differentiation/performance may be attenuated by the up‐regulation of this miRNA in 3T3‐L1. Moreover, systematic modulation of miR‐424(322) in such fat precursors modified growth in proliferating cells before reaching confluence (Figure S4J,K, Supporting Information), which stands for an additional proof of the mechanisms that this tumor suppressor miRNA may control,^[^ ^17^ ^]^ and increased fat cell numbers in our miR‐KO mouse model (Figure S1H,I, Supporting Information). Altogether, in addition to its ability as regulator of cell division, diminished expression of miR‐424(322) is critical during the commitment and terminal differentiation of both mouse and human adipocytes.

### 2.4 Decreased Expression of Adipose miR‐424(322) in Humans Co‐Segregates with Obesity

**Figure 3:**
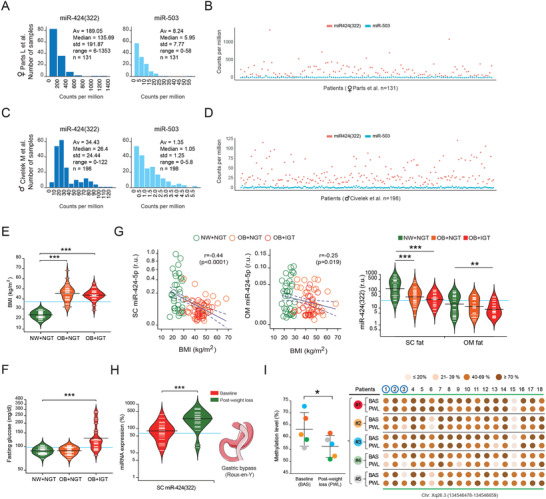
Impaired miR‐424(322) expression in obese human adipose tissue. Raw miRNA counts per million from RNA‐seq performed on adipose tissue from women (Parts et al.^[^ ^47^ ^]^) A,B) and men (Civelek et al.^[^ ^48^ ^]^) C,D), demonstrating that the miR‐424(322) (but not miR‐503) is consistently expressed in human fat, being especially abundant in women (average out at 190 counts per million). Bean plots depict significant variations in E) body mass indexes (BMI) and F) fasting glucose in participants with normal weight (NW) and normal glucose tolerance (NGT) (green; *n* = 34), and in obese (BMI ≥ 30 kg m^−2^) subjects with (red; *n* = 20) and without (orange; *n* = 31) impaired glucose tolerance (IGT). G) Scatter dot plots show the association between subcutaneous (SC) and omental (OM) miR‐424(322) expression levels and BMI, while bean plots represent SC and OM miR‐424(322) in subjects segregated according to their BMI (NW < 30 or obese (OB) ≥ 30 kg m^−2^ particpants) and glucose tolerance. IGT was defined as impaired fasting glucose (≥110 mg dl^−1^) and/or HbA1c of ≥5.7% (see also in Tables S1 and S2, Supporting Information). H) Changes in SC miR‐424(322) expression at the baseline (red) and upon surgery‐induced weight loss (green) in 23 paired pre‐post fat samples (see also in Table S3, Supporting Information), and I) variations in the methylation degree of DNA residues within the miR‐424(322)/503 loci before/after bariatric surgery (*n* = 5). Expression values for miR‐424(322) in bean plots are shown as complementary log‐log (cloglog) distribution. Statistical significance for differences between groups was determined by One‐way ANOVA followed by Tukey's honestly significant difference (HSD) post hoc tests (cross‐sectional sample), and by Student's paired *t*‐test (longitudinal studies). *p < 0.05; **p < 0.01; ***p < 0.001.

Next we transitioned our studies to human samples. To address this, we first assessed the expression levels of miR‐424(322) and miR‐503 in human WAT by re‐analyzing two independent ncRNA high‐throughput sequencing studies consisting of 131 women (median age 58 y; BMI = 27.4 ± 5.5 kg m^−2^),^[^ ^47^ ^]^ and 197 men (median age 57 y; BMI = 26.6 ± 3.3 kg m^−2^).^[^ ^48^ ^]^ In line with previous observations,^[^ ^18^ ^]^ our analysis confirmed an unbalanced miR‐424(322) to miR‐503 expression ratio, whereby miR‐503 was expressed one log of magnitude lower than the miR‐424(322) (**Figure** **3**A–D). This evidence matches our previous real time‐PCR and microarray^[^ ^49^ ^]^ results in differentiating adipocytes, in which expression of miR‐424(322) (presented as relative units of expression, r.u., or log_2_ average intensity) was decoupled from that of miR‐503 (Figure 2C,E, and Figure S3B, Supporting Information). The molecular basis for this biased expression may be explained by a higher miR‐503 turnover caused by the nucleotide composition within its seed region and the 3' miRNA end.^[^ ^50^ ^]^ Interestingly, this molecular instability seems to be a common feature shared by other members of the miR‐15/107 family, and is considered essential in regulating their levels in certain cellular contexts.^[^ ^50^ ^]^

Then, we resolved to study the expression of the cluster miR‐424(322)/503 by real time‐PCR and TaqMan hydrolysis assays in two independent cohorts (Tables S1–S3, Supporting Information). Cohort 1 consisted of 85 individuals (17 men) with a wide range of weight (obesity of 60%) and glucose tolerance (Figure 3E,F, and Table S1, Supporting Information). Cohort 2 included 23 morbidly obese women followed for an average of ≈2 years after bariatric surgery,^[^ ^51^ ^]^ of which resulted in pronounced loss of fat mass and metabolic improvement (Table S3, Supporting Information). In both cohorts, subcutaneous (SC) abdominal biopsies of adipose tissue were taken (for cohort 2, biopsies were taken at baseline and follow‐up). Paired omental (OM) fat samples were also studied in cohort 1. In both SC and OM adipose compartments, miR‐424(322) was strikingly diminished in obese subjects, especially in those with impaired glucose tolerance (IGT) (Figure 3G and Table S1, Supporting Information). Thus, the expression of miR‐424(322) was inversely associated with measures of body mass index (BMI) and fat mass (Figure 3G and Table S2, Supporting Information) independently of potential confounders as age, sex, and the reproductive stats of women (inferred from age) (Figure S5A,B, Supporting Information). Of note, expression of miR‐503 could not be assessed in those fat samples due to undetectable Ct (cycle threshold) values (data not shown), which is consistent with its lower stoichiometric abundance in human adipose tissue (Figure 3A–D). Next, we asked whether expression of miR‐424(322) is altered during major weight loss (mean BMI loss, −33%) resulting from radical reduction in calorie intake after bariatric surgery (Roux‐en‐Y gastric bypass with the purpose of facilitating weight loss). In agreement with our cross‐sectional findings, surgical treatment and the subsequent weight loss resulted in a significant increase of SC miR‐424(322) (Figure 3H and Table S3, Supporting Information). Notably, bisulfite‐sequencing DNA methylation analysis of adipose tissue before/after bariatric surgery (*n* = 5) indicated a significant decrease in methylated residues within the miR‐424(322)/503 loci, which agrees with the increased expression of the miR‐424(322) in obese subjects upon massive weight loss (Figure 3I and Figure S5C, Supporting Information). Altogether, these results provide a body of in vivo qualitative and quantitative evidences that link the expression of the most abundant miR‐424(322)/503 paralog,^[^ ^36^ ^]^ the adipose miR‐424(322), to the burden of obesity in humans.

### 2.5 Transcriptome‐Wide Analysis Reveals That the Cluster miR‐424(322)/503 Tightly Controls the Adipocyte Expression Program

**Figure 4:**
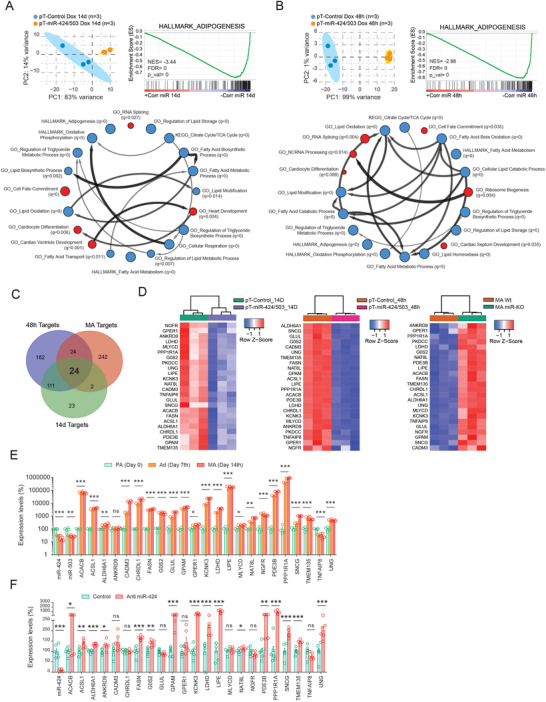
Global transcriptomics reveal that the miR‐424(322)/503 cluster regulates multiple genetic programs related to adipocyte function. RNA sequencing (RNA‐seq) was performed on engineered human adipocytes with increased levels of the cluster miR‐424(322)/503 A) over the course of differentiation (14 days) and B) during the last 48 h of terminal differentiation. Upper left: principal component analysis (PCA). Upper right and down: gene set enrichment analysis (GSEA) was used to uncover enriched gene signatures from the molecular signatures database (MSigDB). Red and blue circles (nodes) represent different annotations exhibiting positive or negative enrichment, respectively (FDR *q*‐val < 0.05). Size of nodes reflect *p*‐value (*p*‐val < 0.05 for all samples). Arrows interconnect pathways presenting common genes in the leading edge subset for each annotation. C) Venn diagram showing the overlap between potential Targetscan (TS) target genes with differential expressions between Wt/miR‐KO mouse adipocytes (red circle); differentiating human adipocytes with or without induction of the miR‐424(322)/503 cluster (green circle); and adipocytes with or without cluster induction for the last 48 h of differentiation (blue circle). D) Heatmaps show hierarchical clustering analysis for the 24‐gene signature predicted to be regulated by the miR‐424(322)/503. Expression of target gene candidates on E) the 0, 7th and 14th day of the course of adipocyte differentiation (*n* = 4/time‐point; One‐way ANOVA), and in F) adipose‐derived mesenchymal stem cells (ASC52telo)‐derived adipocytes challenged with an antagomiR directed against the miR‐424 (*n* = 6/group; Student's *t*‐test). Data are presented as mean ± S.E.M. *PKDCC* did not show qRT‐PCR amplification data. *p < 0.05; **p < 0.01; ***p < 0.001. NS, not significant.

The biological functions of miRNAs are mainly mediated by their ability to attenuate the stability of target messenger (m)RNAs.^[^ ^33^ ^]^ In order to investigate the landscape of genes directly regulated by the cluster miR‐424(322)/503 in adipocytes, we performed deep RNA sequencing (RNA‐seq) on i) undifferentiated MEFs and adipocytes derived from Wt and miR‐KO MEFs; ii) engineered primary human preadipocytes with increased levels of the cluster miR‐424(322)/503, following induction of adipocyte differentiation; and iii) lipid‐containing white adipocytes with induction of the cluster during the last 48 h of terminal differentiation. In line with our computational analysis and in vitro functional assays (Figure 2), gene set enrichment analysis (GSEA)^[^ ^52^ ^]^ provided evidence that the main gene sets enriched in miR‐KO adipocytes (but not in undifferentiated MEFs) were related to fatty acid metabolism and adipocyte differentiation (Figure S6A,B, Supporting Information). On the other hand, an opposite pattern was observed during adipogenesis in human preadipocytes with increased levels of miR‐424(322)/503 throughout the whole adipogenic course (14 d) (**Figure** **4**A), and the same effect was observed in lipid‐filled human adipocytes after an acute induction of miR‐424(322)/503 for 48 h (Figure 4B), thus indicating that the impact of the miR‐424(322)/503 on the adipocyte gene transcriptional program is not exclusively restricted to the blockage of the adipogenic course. Targetscan predictions based on both seed sequence conservation^[^ ^53^ ^]^ and non‐conserved target sites^[^ ^54^ ^]^ were used as a strategy to identify specific miRNA targets among all the messenger RNAs with significant variations (i.e., [FC] > 1.5 plus adj. FDR *p*‐value < 0.05). After integrating our original datasets with computational predictions, we focused on the overlapping genes showing an inverse differential expression pattern between Wt/miR‐KO adipocytes and engineered human adipocytes with persistent/acute increased levels of miR‐424(322)/503 (Figure 4C). Among the interactions identified by this strategy, we found a cross‐species 24‐gene signature characterized by an inverse expression pattern between gain and loss‐of‐function models (Figure 4D and Figure S6C, Supporting Information). Noteworthy, this gene signature was also depicted by increased expression during differentiation of primary human adipocytes (Figure 4E), as opposed to that observed for miR‐424(322) and miR‐503 (Figure 2C), and the majority of the genes included show increased expression levels in adipocytes derived from ASC52telo cells when transfected with miR‐424(322)‐antagonizing oligonucleotides (Figure 4F).

### 2.6 Identification and Validation of Adipogenesis‐Related miR‐424(322)/503 Target Genes

**Figure 5:**
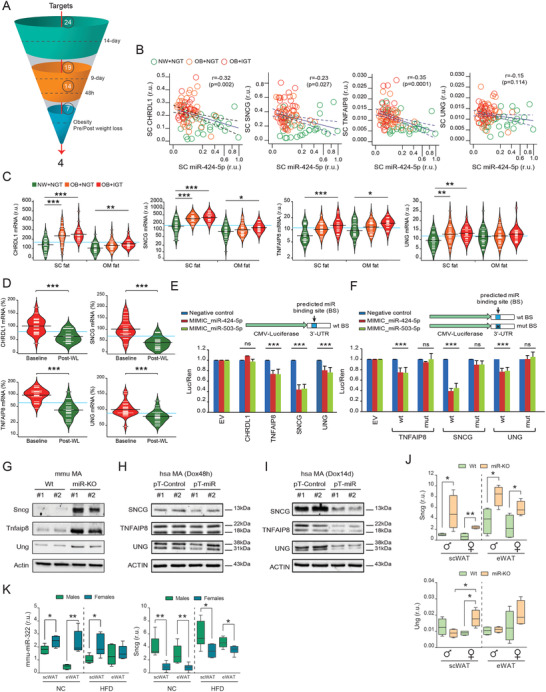
Validation and translational evaluation of miR‐424(322)/503‐regulated target genes. A) The funnel diagram depicts the comprehensive pipeline used to shortlist potential target genes across validation experiments and translational studies. B) Scatter dot plots show the negative correlation between the miR‐424(322) and the expression of the four main miRNA targets, Chordin Like 1 (*CHRDL1*), *γ*‐Synuclein (*SNCG*), TNF Alpha Induced Protein 8 (*TNFAIP8*), and Uracil DNA Glycosylase (*UNG*) in human subcutaneous (SC) fat samples (Spearman's rank‐order correlation tests). C) Bean plots illustrating *CHRDL1*, *SNCG*, *TNFAIP8*, and *UNG* mRNA in normal weight (NW) participants with normal glucose tolerance (NGT) (green, *n* = 34), and in obese subjects without (orange, *n* = 31) and with (red, *n* = 20) impaired glucose tolerance (IGT). D) Expression of SC *CHRDL1*, *SNCG*, *TNFAIP8*, and *UNG* at the baseline (red) and upon surgery‐induced weight loss (green) (*n* = 23). Statistical significance was determined by the Tukey's honestly significant difference (HSD) ANOVA's post‐hoc test (cross‐sectional comparisons), and by paired *t*‐test (longitudinal study) (see also Tables S1–S3, Supporting Information). E) Cloning of the 3'‐UTR regions corresponding to *CHRDL1*, *SNCG*, *TNFAIP8*, and *UNG* and subsequent luciferase assays show differential targeting activity by the miR‐424(322) and the miR‐503, when compared to a control scrambled non‐targeting RNA. Relative light units (*X*–axis) are expressed as the normalized luciferase counts to the renilla internal control. F) Predicted miRNA binding site mutagenesis and luciferase assays were performed in the most relevant candidates (*TNFAIP8*, *SNCG*, and *UNG*) from (E) to prove the miR‐424(322) and miR‐503 binding specificity. Data are presented as mean ± S.E.M. (*n* > 5/group). G) Western blots show that miR‐KO mature adipocytes (MA) have increased gene target protein levels, while H) the 48 h‐lasting induction of the miR‐424(322)/503 cluster triggers a moderate effect on the endogenous levels of adipocyte SNCG, TNFAIP8, and UNG, and I) a more pronounced effect after transient induction with doxycyclin in the 14 day‐lasting adipogenic course assay (*n* = 2/group). Actin immunobloting was used to ensure equal loading protein amounts. J) Messenger *Sncg* and *Ung* RNA expressions in subcutaneous (scWAT) and epididymal (eWAT) white adipose tissue in sex and age‐matched Wt and miR‐KO mice (*Tnfaip8* gene expression was undetectable in mouse fat samples.), and K) mouse miR‐322 and *Sncg* expressions in male and female mice under normal chow (NC) and high‐fat diet (HFD) (*n* = 4/group; Student's *t*‐test). r.u. stands for “relative units.” *p < 0.05; **p < 0.01; ***p < 0.001. NS, not significant.

To understand at a mechanistic level how miR‐424(322)/503 regulates obesity and adipogenesis, we focused on the conserved 24‐target gene signature discovered in our RNA‐seq analysis. We designed a comprehensive pipeline to sequentially filter these genes throughout a series of experimental validations in which we assessed the expression of these genes after induction of the miR‐424(322)/503 with Dox (**Figure** **5**A) i) over the 14‐day adipogenic course (Figure S7A, Supporting Information); ii) during 9 days after completing differentiation (Figure S7B, Supporting Information); and iii) during the last 48 h of terminal differentiation, after which we shortlisted 7 candidates out of the initial 24‐gene set signature (Figure S7C, Supporting Information). Interestingly, these candidates showed decreased gene expression after TGF*β* treatment in preadipocytes and during differentiation, indicating an inverse correlation with the adipogenic process and TGF*β*‐induced miR‐424(322)/503 (Figure S7D,E, Supporting Information). Then, to determine the physiological significance of these miR‐424(322)/503 target genes, we queried the human fat samples of cohorts 1 and 2. Expression levels of as many as 4 target genes (Figure 5A), Chordin Like 1 (*CHRDL1*), *γ*‐Synuclein (*SNCG*), TNF Alpha Induced Protein 8 (*TNFAIP8*), and Uracil DNA Glycosylase (*UNG*) negatively correlated with the expression of miR‐424(322) in subcutaneous adipose tissue (Figure 5B and Table S2, Supporting Information). In addition, opposite to miR‐424(322), *CHRDL1*, *SNCG*, *TNFAIP8*, and *UNG* gene expression levels were upregulated in obese fat samples (Figure 5C and Table S1, Supporting Information) and significantly decreased after surgery‐induced weight loss (Figure 5D and Table S3, Supporting Information).

The biological function of miRNAs is based upon their ability to attenuate the expression of target genes by binding to complementary regions within the 3'UTR.^[^ ^54^ ^]^ To explore the targeting ability of miR‐424(322) and miR‐503 on *CHRDL1*, *SNCG*, *TNFAIP8*, and *UNG*, we cloned the 3'UTR regions of these genes into a luciferase (Luc) reporter system and tracked the ability of synthetic miR‐424 and miR‐503 molecules to modulate Luc activity. As shown in (Figure 5E), the activity of the *CHRDL1* 3'UTR was unaltered by ectopic miRNA treatment, whereas Luc activity for the *TNFAIP8*, *UNG*, and *SNCG* 3'UTR reporters was significantly reduced. Importantly, this inhibitory activity could be abrogated by performing site‐directed mutagenesis of the predicted miRNA binding sites (Figure 5F). Additionally, we assessed whether the 3'UTR targeting activity could also be observed at the protein level. In line with the luciferase reporter assays, western blot studies revealed that the absence of miR‐424(322)/503 in miR‐KO MA was accompanied by a clear increase in SNCG and TNFAIP8 protein levels, with a mild increase in UNG (Figure 5G). Doxycycline‐induced expression of the miR‐424(322)/503 in human MA showed a moderate change in protein levels of target genes after 48 h Dox stimulation (Figure 5H), which progressed into a more consistent effect after 14 days of Dox exposure during differentiation (Figure 5I). Overall, these results point at *SNCG*, *UNG*, and *TNFAIP8*, but not *CHRDL1*, as relevant miR‐424(322)/503 target genes.

To further characterize the relationship between the cluster miR‐424(322)/503 and *Sncg*, *Tnfaip8*, and *Ung*, we also quantified their mRNA expression in Wt and miR‐KO mice. Our gene expression data demonstrated that these correlations were maintained in rodents, especially for *Sncg*. Indeed, male and female mice lacking miR‐424(322)/503 showed increased expression of epididymal and subcutaneous *Sncg* (all genres), and *Ung* (only in subcutaneous fat samples of female mice) (Figure 5J), while *Tnfaip8* gene expression was undetectable (Ct > 40) in mouse fat samples. Notably, measures of gene expression conducted in scWAT/eWAT samples from males and females under a NC or HFD unveiled *Sncg* as the most robust target gene exhibiting an inverse correlation with the miR‐424(322)/503 cluster in mice (Figure 5K), while additional analysis of 3T3‐L1 and ASC52telo‐derived adipocytes respectively challenged with mimic miR‐322 (Figure S7F,G, Supporting Information) and antagomiR‐424 (Figure S7H, Supporting Information) further highlighted SNCG as a bona fide miR‐424(322)/503 target gene in both humans and mice. The above observations also indicate that overall variant expressions of the miR‐424(322)/503 locus in adipocyte progenitor cells, mature adipocytes, or the bulk of adipose tissue may impair SNCG levels, subsequently altering adipogenic commitment and/or fat performance.

### 2.7 The Cluster miR‐424(322)/503 in Adipose Ties Up the Expression and Secretion of SNCG

**Figure 6:**
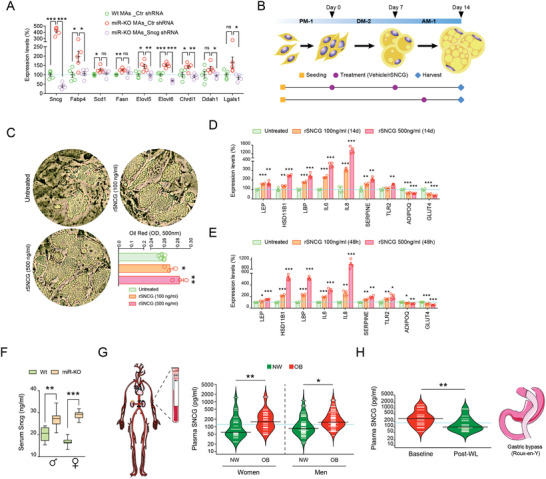
Enhanced secretion of SNCG exacerbates adipogenesis under conditions of decreased miR‐424(322)/503. A) Knock‐down of *γ*‐Synuclein (*Sncg*) in miR‐KO mouse embryonic fibroblasts blunts their enhanced adipogenic commitment, as revealed by gene expression measures in mature adipocytes (MAs; *n* = 5/group; Tukey's “post‐hoc” tests). B) Schematic representation of recombinant (r)SNCG treatments in human pre/adipocytes. PM‐1, DM‐2, and AM‐1 stand for preadipocyte, adipocyte differentiation, and adipocyte maintenance media, respectively. C) Treatments of rSNCG lasting 14 days enhanced the adipogenic commitment of primary fat precursor cells, as revealed by Oil Red O staining, and either(D) sustained (during the adipogenic course) or E) acute (48‐h exposure in fully‐differentiated adipocytes) treatments dose‐dependently (i.e., 100 and 500 ng mL^−1^) increased expression signatures suggestive of an altered inflammatory activity. Data are presented as mean ± S.E.M (*n* > 3/group). Tukey's “post‐hoc” tests were used to determine significant differences versus control). F) Serum SNCG levels in Wt and miR‐KO males and females under NC (*n* > 6/group; Sudent's *t*‐test). G) Bean plots show variations in plasma SNCG for non‐obese (BMI<30 kg m^−2^; *n* = 76, green) and obese (*n* = 102, red) women and men, and H) in obese patients (*n* = 19) at the baseline (red) and upon ≈1 years of surgery‐induced weight loss (green). Statistical significance was assessed by paired Student *t*‐tests (see Table S4, Supporting Information for additional details). *p < 0.05; **p < 0.01; ***p < 0.001. NS, not significant.

Thus far, we provide in vivo and in vitro data to establish miR‐424(322)/503 as a previously unexplored mediator of adipogenesis via control of at least three target genes. Among these, *SNCG* was most consistently affected by miR‐424(322)/503 manipulations in both humans and mice. Others have shown that SNCG is involved in mediating metabolic functions in fat cells,^[^ ^20^ ^]^ allowing adipocyte differentiation and fat mass expansion in conditions of nutrient excess.^[^ ^21^ ^]^ Thus, this miR‐424(322)/503 target gene may have particular cellular and physiological implications in the obesity arena through regulation of *SNCG*. To determine whether the effects of miR‐424(322)/503 loss on adipogenesis were directly linked to *Sncg* and changes in the transcriptional program of differentiating adipocytes, we infected miR‐KO MEFs with lentiviruses expressing either silencing short hairpin (sh)RNAs against mouse *Sncg*, or a non‐silencing (NS) control. Noteworthy, reducing *Sncg* was sufficient to reduce differentiation of miR‐KO MEFs into adipocytes, as measured by gene expression of well‐recognized adipogenic markers (**Figure** **6**A). Although the molecular understanding of how SNCG exerts its function is far from complete, it has been shown that SNCG protein is secreted to the extracellular space, where it may act as a paracrine signal that mediates cell‐to‐cell crosstalk.^[^ ^55^, ^56^ ^]^ Thus, to complement our functional studies we applied recombinant SNCG (rSNCG) to human preadipocytes following the adipogenic course, and also performed treatments of 48 h in differentiated adipocytes (Figure 6B). Noteworthy, fat cells challenged with exogenous SNCG displayed increased adipogenic potential (Figure 6C) and showed expression patterns suggestive of hypertrophy, impaired metabolism, and altered inflammatory activity (Figure 6D,E).

Finally, we took advantage of recent evidences demonstrating that SNCG can be secreted into body fluids and investigated as a cancer biomarker.^[^ ^57^ ^]^ Thus, we used an enzyme‐linked immunosorbent assay (ELISA) to measure plasma levels of SNCG in our miR‐KO mouse model. A steadily higher level of circulating Sncg was identified in miR‐KO plasma, when compared to Wt animals of the same sex and age (Figure 6F). Taking into account significant variations in weight and fat mass contributing to the expression of miR‐424(322) and *SNCG* in human adipose tissue, we also analyzed circulating SNCG levels in subjects with different degrees of obesity. Thus, we queried two additional cohorts (Table S4, Supporting Information). Cohort 3 consisted of 178 individuals (55 men) with (57%) or without obesity, defined as BMI ≥ 30 kg m^−2^. Cohort 4 included 19 morbidly obese patients (6 of them men) followed for an average of ≈1 year after bariatric surgery. Intriguingly, our data revealed that plasma SNCG levels were increased in obese subjects (Figure 6G), and normalized concomitantly with surgery‐induced weight loss (Figure 6H). Overall, these results reinforce the functional relevance of impaired SNCG, which impacts the adipose content by enhancing adipocyte differentiation and inflammatory gene expression patterns suggestive of a hypertrophied and dysfunctional fat cell phenotypes.

## 3 Discussion

Since the discovery in 1993 of the first microRNA (miRNA),^[^ ^58^ ^]^ miRNAs number, function, and biological relevance have increased exponentially in almost all science fields: from the control of leaf and flower development to human reproduction, metabolism, cancer, or degenerative diseases. However, in comparison with other biomedical areas, the field of miRNA genetics has traditionally lacked clear translational impact because of a mixed combination of factors: the paucity of animal models with a noticeable phenotype, the inability to overcome the cross‐species (mouse‐human) conservation gap, and by the limited biological relevance and scarce information obtained from human samples. In the present study we present molecular, cellular, and physiological evidences demonstrating that miRNAs are relevant for the homeostatic balance of adipose tissue and that its altered expression can be related to metabolic complications in humans. Specifically, we are the first to outline a novel role for the cluster miR‐424(322)/503 in regulating the adipocyte transcriptional program, adipocyte differentiation, and the bulk of adipose tissue mass in both mice and humans. Our data demonstrate that modulation of miR‐424(322)/503 expressions in adipocytes impacts the adipogenic commitment and differentiation of these cells, as shown by multiple in vitro cell assays and global transcriptomics. Concomitantly, loss of the cluster miR‐424(322)/503 in mice promotes adipose tissue expansion and alters early progenitor homeostasis, as shown by gross physiological and histological analysis and population‐based flow cytometry studies. It must be noted, however, that since our data is partially based on a global knockout mouse model, the contribution of non‐white adipose effects on adiposity and altered energy balance in the miR‐KO mouse cannot be ruled out. In this regard, neither differentiation of brown adipocytes^[^ ^59^, ^60^ ^]^ nor cold exposure^[^ ^61^ ^]^ or HFD‐induced obesity^[^ ^62^ ^]^ modify the expression of our miRNA candidates (Figure S8A–C, Supporting Information) or mouse (m)H19X, the lncRNA host gene containing the miR‐424(322)/503 cluster in mice^[^ ^63^, ^64^ ^]^ (Figure S8D,E, Supporting Information), and no significant modulation of brown performance/activity marker genes in brown adipose tissue (BAT) was identified in miR‐KO mice (Figure S8F, Supporting Information), thus decoupling to some extent the obese phenotype of our mouse model from defective BAT activity. While we did not explore the biological role of this miRNA cluster in other systems, tissue distribution of miR‐424(322)/503 expressions in mice,^[^ ^18^ ^]^ the curated analysis of its target gene expressions in fat depots, and our complementary cellular models and analysis of transversal/longitudinal human patient cohorts indicate, altogether, that this miRNA cluster (namely the miR‐424(322) component) plays a key role in the bulk of WAT and lipid homeostasis, at least in a cell autonomous manner. Our data also indicate that adipose miR‐424(322) expression inversely correlates with human obesity and the expression of the target genes identified along this research. Our findings demonstrate that the cluster miR‐424(322)/503 regulates the fatty acid gene expression program in adipocytes, and we provide abundant evidence that this regulation is mainly mediated through the control of *γ*‐Synuclein (SNCG), a regulatory factor in fat cells that contributes to the development of obesity by promoting lipid metabolism and fat mass expansion.^[^ ^20^, ^21^ ^]^

Our findings also support the contention that expression of the cluster miR‐424(322)/503 is prompted by transforming growth factor beta (TGF*β*),^[^ ^18^, ^43^ ^]^ particularly in preadipocytes. TGF*β* is known to yield a strong inhibitory effect on the adipogenic commitment of preadipocyte cell lines^[^ ^44^ ^]^ and primary cell cultures.^[^ ^65^ ^]^ TGF*β* blocks adipogenesis in vivo, as transgenic overexpression of TGF*β* in adipose tissue severely reduces depots of adipose tissue, whereas adipocytes fail to differentiate.^[^ ^66^ ^]^ Our results confirm that TGF*β* signaling depletes the rate of adipogenesis in vitro and unveil miR‐424(322)/503 as a key player in the endogenous control of differentiating fat cells, demonstrating the cluster's negative influence on adipocyte differentiation, even if its absence does not restrain the adipogenesis‐repressive/reversible effects of TGF*β* (Figure S8G,H, Supporting Information). We found that the loss of the cluster miR‐424(322)/503 enhanced adipogenesis, which correlated with the increased ability to generate white hypertrophied adipocytes. On the other hand, the loss of miR‐424(322) in obese subjects may be due to the refractory response to TGF*β* that may depict fully‐differentiated adipose cells,^[^ ^67^ ^]^ the hormonal milieu,^[^ ^68^, ^69^ ^]^ and/or altered mH19 imprinting in obese subjects (Figure 3I). However, because our cross‐sectional sample was not population‐based, we cannot exclude that the observed relationship is different in specific subgroups (e.g., lean and obese subjects, men and women). Nevertheless, these results were not dependent on other clinical phenotypes according to multivariate analyses, and observations made in obese subjects following massive weight loss further support the relationship between abnormally low miR‐424(322), enhanced target genes expression, and the burden of obesity. The exact mechanism whereby thinness, obesity, and surgery‐induced weight loss may influence the expression of the cluster miR‐424(322)/503 in fat is a key question we seek to answer in our future studies. However, current evidence strongly suggests that this expression underlies the silencing of genes closely related to the differentiation of fat cells and the development of an obese phenotype in both mice and humans.

Here, we defined the endogenous miR‐424(322)/503 target network in mouse and human adipocytes and found significant variations in complementary approaches. Our combined results establish that miR‐424(322)/503 constitutes an as yet unexplored layer in adipogenesis regulation. The cluster may also promote the “transcriptome shift” from mature adipocyte to partial reversion back to the precursor cell state, as outlined by engineered human adipocytes challenged with increased levels of miR‐424(322)/503 for 2 and 9 days. Unambiguous target sites of these broadly conserved miRNAs were found throughout major genomic annotations and encompassed adipogenesis‐bulged binding sites. Yet, only three 3'UTRs found in canonical miR‐424(322)/503 target sites had a significant impact on their respective steady‐state mRNA and protein levels, as shown through complementary in vitro and in vivo observations, and luciferase assays. Our comprehensive studies identified *SNCG* as a robust target gene of the miR‐424(322)/503 cluster in adipose tissue and adipocytes, both in mice and humans. This synuclein family member, also known as breast cancer‐specific gene (BCSG1),^[^ ^70^ ^]^ Synoretin,^[^ ^71^ ^]^ and Persyn,^[^ ^72^ ^]^ has been predominantly associated with various forms of cancer, as a predictive and diagnostic value, as a marker for assessing tumor grade, and as a potential therapeutic target.^[^ ^73^ ^]^ High levels of *SNCG* gene expression have been also reported in adipose tissue, namely in mature adipocytes. This expression is more prominent in obese individuals and situations where adipose plasticity and adipocyte turnover may be engaged.^[^ ^21^ ^]^ In 2012, Millership et al.^[^ ^20^ ^]^ set out to define the specific role of SNCG in fat mass expansion by means of a *Sncg*‐null mice following a high fat‐diet. They observed that the loss of *Sncg* protects against the development of diet‐induced obesity, while ameliorating obesity‐related hyperinsulinemia and hepatosteatosis. In the current study we show that adipose tissue and adipocytes from our miR‐KO mouse model have steadily increased levels of *Sncg*, coupled with the higher amounts of this protein in circulation. Lower expression of miR‐424 in obese subjects correlated with increased levels of SNCG in human fat debris and blood samples in cross‐sectional studies, and in morbidly obese patients following significant weight loss by means of surgical procedures. As results in engineered and human adipocytes challenged with antagomiR‐424 imply that acute/long‐lasting up‐regulation of the miR‐424(322)/503 cluster (namely the miR‐424(322) component) specifically and cell‐autonomously reduces SNCG, additional experiments performed during this research show the unexpected impact of this synuclein on fat cell differentiation and adipocyte commitment when exogenously administrated. These findings raise the possibility that the cluster miR424(322)/503 may have a fundamental function in fat depots by controlling both SNCG‐mediated profusion of hypertrophied and inflamed adipocytes, and the secretion of this messenger to the media. Of note, overexpression of SNCG has been associated with diseases to which obese individuals are more prone (e.g., esophageal, colorectal, prostate, and, in particular, breast cancer, but also some neurodegenerative disorders).^[^ ^74^ ^]^ Considering this, along with the pioneering impact of exogenous SNCG on fat cells and circulating measures taken in obese and lean subjects, further investigations and assessment in additional cellular systems and clinical samples are warranted.

Numerous studies have identified miRNAs expressed in fat depots, being closely related to the adipogenic course, that aimed to stimulate or inhibit the differentiation of white adipocytes, and to orchestrate key metabolic and endocrine functions.^[^ ^75^ ^]^ However, little is known about the role of miRNAs in the control of fat cell size and function in the burden of obesity. The physiological significance and clinical relevance of miRNAs are often uncertain due to the unavailability of complementary data from engineered mouse models and patient sample analysis. Additionally, while therapeutic modulation of miRNAs provides a promising approach for obesity and impaired metabolism, the promiscuous nature of miRNAs raises many concerns over detrimental off‐target effects and the translational utility of experimental results. Systemic dissection of these connections, especially at molecular and organism levels, would likely yield important insights that are currently unavailable. The H19X‐encoded miR‐424(322)/503 cluster regulates fundamental cellular processes that include cell proliferation, cellular plasticity, epithelial‐to‐mesenchymal transition, stress response, metabolic clues, and tissue differentiation and remodeling.^[^ ^17^ ^]^ In this work, we present a framework endorsing a previously unknown biological function of the cluster miR‐424(322)/503. This novel role is supported by an overlap of functional in vivo results, as well as an exhaustive assessment of complementary cell systems and clinical data disclosing the translational validity of our experimental observations. Altogether, our study highlights a novel mechanism that regulates the differentiation and commitment of fat cells. These novel findings shed light on the physiological and molecular mechanisms involved in the occurrence and development of obesity.

## 4 Experimental Section

### Animals

Generation and genotyping of miR‐424(322)/503^−/−^ mice on a mixed C57BL/6 plus BALBc genetic background had been described previously.^[^ ^18^ ^]^ Briefly, double miR‐424(322) and miR‐503 gene editing were engineered using the ZiNc Finger nuclease technology (ZNF, Sigma Aldrich). Two ZNFs were used for each miRNA to cut upstream and downstream of selected sequences. ZNFs were injected into 400 fertilized oocytes, which were in turn implanted in recipient C57Bl6J female mice. Primers used for genotyping were pair 1 (Fwd, CACCAGCAGATCCTGGAAAT; Rev, CAAGTGAGGCGCTAACAACA) and pair 2 (Fwd, AGTTTCGAAAAACGGACATA; Rev, ATTGCCTCTCATTGTACCAC). To address all experimentation included in this manuscript, the C57BL/6 plus BALBc/miR‐424(322)/503^−/−^ model was first crossed with wild‐type C57BL/6 mice for more than ten generations to produce a >99.5% clean C57BL/6/miR‐424(322)/503^−/−^ background. Phenotyping of wild type (Wt) and miR‐424(322)/503^−/−^ (miR‐KO) virgin male and female mice in reproductive age (3 months) on either regular NC (Special Diet Services, Dietex, RM3(E) irradiated to 2.5 Mrads; 12.1 kJ g^−1^; 4% fat, 48% carbohydrate, and 14% protein) or 60% HFD (Research Diets, Inc., D12492; 60% kcal from fat, 20% from protein, and 20% from carbohydrate) was performed at the MRC Harwell Institute (Mary Lyon Centre, Harwell Campus, Oxfordshire, OX11 0RD, UK. http://www.har.mrc.ac.uk). All procedures were conducted in accordance with the Animals Scientific Procedures; Act 1986, Amendment Regulations 2012 (SI 4 2012/3039). Drinking water consisted of mains water through reverse osmosis filter and chlorinated to 9–13 ppm. Bottles were changed weekly with autoclaved bottle and cap. NC or HFD was administered to mice at 6 weeks of age until week 19. The metabolic phenotype was assessed between weeks 16 and 18 of age. Phenotyping included monitored growth and daily weight measurements, MRI, and blood chemistry (determination of glucose, cholesterol, triglycerides, and FFA by means of commercially available kits) for both pipelines.

### Glucose Tolerance Testing

IPGTT were performed in 19 week‐old animals fasted overnight. Blood glucose was measured by Accu‐Chek Active glucometer (Roche diagnostics). For glucose tolerance test, 2 g glucose per kg was administered intraperitoneally, and blood glucose was measured at 0, 15, 30, 60, 90, and 120 min from tail tip. The areas under the glucose curves were evaluated according to the trapezoidal rule in each animal.

### Adipocyte Numbers

Subcutaneous and epidimal depots of WAT were fixed in formalin and scanned using a structured white‐light 3D scanner with an accuracy of 0.05 mm (Range Vision NEO Scanner, RangeVision, Russia). Samples were disposed on an automatic rotary table and sprayed with AESUB spray (Scanningspray Vertriebs UG, Germany) to improve scannability. In each scanning process, the rotary table scanned the sample in 12 different positions. After a first scan was performed, the sample was turned on the table to ensure all areas from the sample were adequately scanned, including the part of the sample that was in contact with the rotary table in the first scan. Later, the two scans of each sample were integrated using the native software of the scanner (RаngeVisiоn SсаnCenter, RangeVision, Russia). This resulted in a 3D mesh file of each sample, which was processed, and volume measured using Mesh Lab, an open‐source tool for the visualization and processing of 3D models.^[^ ^76^ ^]^ Fat samples were then embedded in paraffin and some sections were stained with hematoxylin and eosin (H&E). The area of 100 adipocytes in 10× images of fat per mice was measured using the ImageJ software (Rasband, W. S., National Institutes of Health, Bethesda, Maryland, USA). To calculate the average adipocyte volume, the radius of each individual adipocyte area was estimated, assuming a circular model. The estimated average radius was then used to calculate the volume (assuming a spherical model) of each adipocyte by means of the sphere volume formula: *V* = 4/3*π*r^3^. Number of adipocytes was finally provided as volume of fat deposit/average volume of adipocytes.^[^ ^77^ ^]^

### Adipocytes’ Size

To determine the effects on adipocyte morphology, visceral and subcutaneous fat was obtained immediately after euthanasia. Fat pads of mice from each group were fixed in formalin and embedded in paraffin, and cut in 5 micrometer sections, and stained with H&E. ImageJ was used within 10× images to analyze the area of 100 adipocytes. Cells were outlined by enhanced contrast function and the calculated area was obtained, in pixels, for each as described in Parlee and colleagues.^[^ ^77^ ^]^ This process was applied to 5 images per slice, resulting in 1500 adipocytes analyzed per genotype and sex or 500 adipocytes per mouse. Measurements were obtained from three individual animals per group. Data are presented as the average ± SD for each group. Gaussian fit histograms showing adipocyte area distribution were calculated as described in.^[^ ^78^ ^]^

### Human Samples

This study was also intended to examine the expression of the miR‐424(322)/503 locus in human adipose tissue. Hence, measures of miR‐424‐5p and miR‐503‐5p in abdominal subcutaneous fat samples were retrieved from publicly available GEO datasets (http://www.ncbi.nlm.nih.gov/geo/) and ArrayExpress (https://www.ebi.ac.uk/arrayexpress/), with accession numbers GSE45159^[^ ^48^ ^]^ and E‐TABM‐1140,^[^ ^47^ ^]^ respectively. The expression profiling of these datasets was based on Illumina platforms. Series matrix files were downloaded to screen and verify miR‐424(322) and miR‐503 expression levels in human adipose tissue. GSE45159 collected 197 fat samples from Finnish men aged 57 ± 7 years (BMI = 26.6 ± 3.3 kg m^−2^), and E‐TABM‐1140 contained a final amount of 131 participants (only women; median age 58 y; BMI = 27.36 ± 5.53 kg m^−2^). The samples in these two databases were collected with needle biopsies in the population of the Kuopio town, in eastern Finland,^[^ ^79^ ^]^ and by means of punch biopsies taken in volunteers of the UK Adult Twin Registry,^[^ ^80^ ^]^ respectively. Expression of miR‐424(322) was also assessed in a total of 170 fat samples obtained from omental (OM) and subcutaneous (SC) fat depots during elective surgical procedures (i.e., cholecystectomy, surgery of abdominal hernia, and gastric bypass). Samples of adipose tissue were provided from 17 men and 68 women (*n* = 85) with BMI between 20 and 70 kg m^−2^. Twenty of the 51 participants with obesity showed either impaired fasting glucose (100–125 mg dL^−1^), or HbA1c of 5.7% to 6.4%, both criteria for IGT. Fat samples were washed in phosphate‐buffered saline solution (PBS), fragmented, and snap frozen in liquid nitrogen before being stored at −80 °C. The study protocol was approved by the Ethics Committee and the Committee for Clinical investigation (CEIC) of the “Hospital Universitari Dr. Josep Trueta de Girona.” Samples of SC adipose tissue after surgery‐induced weight loss were also analyzed. Around 2 years after bariatric surgery, an incisional biopsy of SC adipose tissue from the abdomen was obtained. This study is described in detail elsewhere.^[^ ^51^ ^]^ All subjects were of Caucasian origin, gave written informed consent after the purpose of the study was explained to them, and reported that their bodyweight had been stable for at least 3 months before entering the study. Samples and data from subjects included in this study were provided by the FATBANK platform, promoted by the CIBEROBN, and coordinated by the IDIBGI Biobank (Biobanc IDIBGI, B.0000872), integrated in the Spanish National Biobanks Network. All samples were handled in accordance with the standard operating procedures and the approval of the Ethics, External Scientific, and FATBANK Internal Scientific Committees.

### Cell Cultures

MEFs were isolated from Wt and miR‐424(322)^−/−^/503^−/−^ (miR‐KO) E14 embryos. First, head, limbs, and intestinal organs were removed. Then, E14 embryos were cut into small pieces on a plate with PBS, transferred into a falcon with 0.25% Trypsin‐EDTA (Gibco, 25300‐054), and incubated 15 min at 37 °C. Then, the digested mixture was transferred into a new tube and centrifuged to eliminate non‐disaggregated tissue at 200 rpm for 3 min. Cell density was measured using trypan blue (Sigma, T8154). Supernatants were diluted, plated, and cultured with DMEM (Gibco, 31966047), 10% FBS (Gibco, 10270‐106), and 50 IU mL^−1^ penicillin/streptomycin (Gibco, 15070063). Adipogenesis was induced in 2 days post‐confluent miR‐KO and Wt MEFs by means of 0.5 mm 3‐isobutyl‐1‐methylxanthine (IBMX; Sigma, I7018), 1 µm dexamethasone (Sigma, D1756), 5 µg mL^−1^ insulin (Sigma, I3536), and 0.5 µm rosiglitazone (Acros Organics, 462410010) in DMEM containing 10% FBS. On day 4 and thereafter, IBMX, dexamethasone, and rosiglitazone were removed, and 5 µg mL^−1^ insulin was maintained for 4 additional days. Several vials of cryopreserved subcutaneous preadipocytes (SP‐F‐1) from Caucasian women of approximately same age and BMI and commercially available preadipocyte's culture and differentiation media were purchased (Zen‐Bio, Inc.). To induce differentiation, confluent preadipocytes in preadipocyte medium (PM‐1) were incubated with adipocyte differentiation medium (DM‐2) for 7 days, according to manufacturer's instructions. This media was composed of DMEM/Ham's F‐12 (1:1), HEPES, FBS, biotin, pantothenate, insulin, dexamethasone, IBMX, PPAR*γ* agonist, penicillin, streptomycin, and amphotericin B. Thereafter, adipocytes were maintained in adipocyte maintenance medium (AM‐1) for 7 days (DM‐2 without dexamethasone, IBMX, and PPAR*γ* agonist). During this process, the shape of preadipocytes evolved from the flattened form to rounded cells containing abundant lipid droplets, and was thus considered mature adipocytes (12th day and thereafter). Beyond day 14, differentiated adipocytes were maintained in adipocyte basal medium (BM‐1), which contains the components necessary to support stability and activity of mature adipocytes (DMEM/Ham's F‐12 (1:1), HEPES, biotin, and pantothenate). To check whether stimuli that may compromise adipocyte differentiation may lead to reciprocal changes in the expression of the miRNA candidates, human preadipocytes were also differentiated with adipocyte differentiation and maintenance media containing 10 ng mL^−1^ of recombinant human TGF*β* (R&D Diagnostics, 240‐B). One hundred or 500 ng mL^−1^ of recombinant human SNCG (Signalway Antibody LLC, AP70791) were also applied to human preadipocytes differentiating toward mature adipocytes (treatments added together with both adipocyte differentiation and maintenance media), or to lipid‐filled differentiated adipocytes (48 h). For the main purpose of additional testing and validation of previous results in primary cells, human telomerase reverse transcriptase‐immortalized adipose‐derived mesenchymal ASC52telo stem cells (SCRC‐4000, ATCC, LGC Standards SLU, Barcelona, Spain)^[^ ^46^ ^]^ were cultured in a Mesenchymal Stem Cell Basal Medium (ATCC, PCS‐500‐030) plus FBS (2%), recombinant human (rh)FGF basic (5 ng mL^−1^), rhFGF acidic (5 ng mL^−1^), rhEGF (5 ng mL^−1^), L‐Alanyl‐L‐Glutamine (2.4 mm) and G418 (0.2 mg mL^−1^) at 37 °C in a 5% CO2 in air atmosphere. 24 h after plating, cells were checked for confluence, and differentiation was induced by means of the commercially available DM‐2 and AM‐1 media as explained above. 2 weeks after initializing differentiation, differentiated cells appeared rounded with large lipid droplets apparent in their cytoplasm. The embryonic fibroblast mouse 3T3‐L1 cell line (ATCC, CL‐173) was cultured in DMEM containing 4.5 g L^−1^ glucose, 10% FBS, 100 U mL^−1^ penicillin, and 100 µg mL^−1^ streptomycin. 3 days after confluence, a mixture of insulin (1 µg mL^−1^), dexamethasone (0.25 µm), and isobutyl methylxanthine (500 µm) was added to the media for 4 days, followed by 4 days with 1 µg mL^−1^ insulin alone. After 8 days of differentiation, these cells evolved in differentiated lipid‐containing adipocytes.

### Models of Gain and Loss‐of‐Function

Conditional doxycycline (Dox)‐inducible expression of the cluster miR‐424(322)/503 was accomplished by means of lentiviral particles. These constructs were obtained by transfecting phoenix‐packaging cells (ATCC, CRL‐3213) with linear‐jetPEI (Polyplus, 101–10N), in combination with control pTRIPz or the previously reported pTRIPz‐miR‐424(322)/503 system,^[^ ^18^ ^]^ and the pCMV‐dR8.91 and pMD.G helper plasmids^[^ ^81^ ^]^ at a ratio of 2:1:1, respectively. 24 h after transfection, media supernatants were collected and lentiviral particles were concentrated using centrifugation columns (Sigma, UFC910024). Transduction of human preadipocytes with lentiviral particles carrying pTRIPz constructs was conducted as follows: pre‐confluent (50–70%) preadipocytes were incubated in PM‐1 containing pT‐miR‐424/503 or pT‐Control lentiviral particles (2:1) and 10 µg mL^−1^ Polybrene (Sigma, TR‐1003) for 24 h. 24 h after, positive preadipocytes harboring these constructs were enriched by 2 ng mL^−1^ Puromycin dihydrochloride (Sigma, P9620). To induce increased expression of miR‐424(322) and miR‐503, doxycycline (Alfa Aesar, J60579) was added to the culture media at 500 ng mL^−1^ of final dilution for the indicated periods of time (see in Figure 2I cell culture and treatment pipelines). Permanent silencing of *γ*‐Synuclein (*Sncg*) was achieved in miR‐KO MEFs by transducing cells (2:1) with *Sncg* sh‐RNA (Santacruz, SC‐42290‐V), or control non‐silencing (NS) lentiviral particles (Santacruz, SC‐108080). 48 h post‐transduction, properly transfected cells were selected and enriched by means of 3 ng mL^−1^ puromycin before reaching confluency and inducing differentiation. ASC52telo and 3T3‐L1 cells were respectively transfected with miRNA‐antagonizing oligonucleotides and mimic miRNAs along the course of differentiation (sustained treatment in each cell culture media exchange) and in differentiated adipocytes (treatment of 96 h in adipocyte maintenance media). The single mimic and double stranded antagomiRs used for this research were the following (both from Horizon Discovery, Waterbeach, UK): synthetic miRIDIAN Hairpin Inhibitor against human has‐miR‐424‐5p (IH‐300717‐07‐0010), and a synthetic miRIDIAN‐Mimic of mice mmu‐miR‐322‐5p (C‐310534‐05‐0010). Briefly, either mimicking or antagonizing oligonucleotides and Lipofectamine RNAiMAX (Life Technologies, Darmstadt, Germany) were diluted and mixed together with Opti‐MEM I Reduced Serum Medium (Life Technologies, Darmstadt, Germany). Complexes were left to incubate for 20 min at room temperature and added drop‐wise to the adherent cells. Final concentration was 50 nm in 1:2 (RNA:RNAiMAX) ratios. miRIDIAN microRNA Mimic Negative Control #1 (77CN‐001000‐01‐05) and miRIDIAN microRNA Hairpin Inhibitor Negative Control (77IN‐001005‐01‐05) were used as negative reference non‐targeting controls. For each assay, intracellular lipid accumulation was measured using Oil Red O staining. When Oil Red O staining was not available, lipid droplet area was quantified by means of the Fiji software (https://imagej.nih.gov/ij/), a platform for custom biological image analysis.^[^ ^82^ ^]^ Image processing approaches were applied to the area taken by lipid droplets, which were quantified using a threshold plugin after sharpening pixel intensity. Values obtained were expressed as the percentage (%) of total microscopy image filled by highly refringent vacuoles of lipid within the cytoplasm of adipocytes observed under bright‐field illumination. Three to eight biological replicates were collected separately for total DNA, RNA, and protein extraction after adipocyte differentiation and treatment, if any. Gene expression measures were assessed by real time PCR.

### RNA‐seq

Complete RNA‐sequencing data that support the findings of this study have been deposited in the community‐endorsed repository Gene Expression Omnibus (GEO) (http://www.ncbi.nlm.nih.gov/geo/) with the accession code number GSE185819. 2 × 75bp paired‐end polyA capture RNA sequencing was performed on a NextSeq500 Illumina sequencing platform at MAR Genomics (http://sam.imim.es). Raw sequencing reads (coverage 30M reads/sample) in the fastq files were mapped with STAR version 2.5.3a^[^ ^83^ ^]^ to the Gencode release 29 based on the GRCh38.p12 reference genome (for human samples), and to the Gencode release 17 based on the GRCm38.p6 reference genome (for mouse samples), together with corresponding GTF files. Table counts were obtained with FeatureCounts function in the package subread, version 1.5.1.^[^ ^84^ ^]^ The differential expression genes (DEG) analysis for different conditions was assessed with a variance modeling at the observational level (voom) plus an empirical Bayes moderated t‐statistics model (limma) in the limma package version 3.30.13^[^ ^85^ ^]^ and the R (version 3.3.2) programming environment (https://www.R‐project.org/), together with different Comprehensive R Archive Network and Bioconductor packages (http://cran.r‐project.org/). Genes having less than 10 counts in at least 3 samples were excluded from the analysis. Raw library size differences between samples were treated with the weighted trimmed mean of *M*‐values normalization method (TMM), implemented in the edgeR package.^[^ ^86^ ^]^ These normalized counts were used in order to make the unsupervised analysis, PCA, and clusters, also used to discard any potential batch effect. For the differential expression (DE) analysis, read counts were converted to log2‐counts‐per‐million (logCPM) and the mean‐variance relationship was modeled with precision weights using voom approach in limma package. Linear models in a paired design were applied to obtain differentially expressed genes between studied conditions. Then, correction for multiple comparisons was performed using false discovery rate (FDR),^[^ ^87^ ^]^ and adjusted p‐values were obtained. Finally, GSEA was performed with lists of probes showing significant changes with regard to respective controls. The FDR *q*‐value score of 0.05 was set as a threshold. A pre‐ranked metric analysis was performed using the log‐fold change following the guidelines set by the Broad Institute (https://software.broadinstitute.org/gsea/doc/GSEAUserGuideFrame.html). Three collections of gene sets were evaluated: Hallmark, C2 (containing all curated gene sets), and C5, which includes the gene ontology (GO) biological process. Pathways diagrams were performed using Enrichment Map application in Cytoscape (v.3.7.2).^[^ ^88^ ^]^ Relative miR‐424 and miR‐503 abundance in humans was achieved by re‐analyzing two independent ncRNA‐seq cohorts performed in men and women.^[^ ^47^, ^48^ ^]^ miRNA counts data were downloaded and miR‐424 and miR‐503 counts were normalized in relation to the sum of all the miRNA counts for the corresponding individual (Counts Per Million), following previous recommendations.^[^ ^89^ ^]^ Cross‐species human/mouse miR‐424(322) and miR‐503 targets were identified by downloading and integrating predictions from Targetscan_V7.2. Targets were further filtered by their expression (RPKM/TPM > 0.5) in human/mouse adipose tissue. Briefly, target RKPM values for human adipose tissue were downloaded from^[^ ^38^ ^]^ and fastq files from mouse subcutaneous/genital fat tissue were downloaded from^[^ ^37^ ^]^ and TPM values were calculated using SALMON.^[^ ^90^ ^]^ GO term enrichment was achieved by using FatiGO.^[^ ^91^ ^]^ Differentially expressed miRNAs between wild type and Dgcr8 knockout BAT were extracted from^[^ ^60^ ^]^ and represented in a scatter plot. Those miRNAs with fold change >[2.23] were highlighted. MiRNA data from mature brown adipocytes and BAT preadipocytes were downloaded from ref. [59]. Following the study analysis, miRNAs with an absolute fold change >[2] were highlighted in a scatter plot. Raw BAT miRNAs intensity data from mice at room temperature or after cold exposure at 8 °C for 24 h were downloaded from^[^ ^61^ ^]^ and processed with AgiMicroRna R package^[^ ^92^ ^]^ using TGS protocol with default settings. A scatter plot of normalized miRNAs intensity between the two conditions was performed and, following the study analysis, miRNAs with fold change >[1.2] were highlighted. For the analysis of lncRNA expression in BAT, FPKM gene expression from mice chronically exposed to control diet versus high fat diet or mice housed at 22 °C versus 4 °C for 24 h were downloaded from^[^ ^62^ ^]^ and represented in two scatter plots (fold change >[2]). Normalized miRNA intensity from human WAT preadipocyte data was downloaded from^[^ ^49^ ^]^ and the average value between biological replicates was calculated. Log2 values from both lean and obese subjects on day 0 were plotted comparing with differentiation days 7 and 14. MiRNAs with an absolute fold change >[1.2] were highlighted. All statistical analyses and graphical representations were conducted in R version 3.6.2.^[^ ^93^ ^]^

### Gene and miRNA Expressions

Total RNA was purified using the RNeasy Mini Kit (QIAgen, 74104). Concentration was measured by means of a NanoDrop 1000 Spectrophotometer (Thermo Scientific). For gene expression, 3 µg of RNA was reversed transcribed to first‐strand cDNA using the High Capacity cDNA Archive Kit (Applied Biosystems, 4368814). For miRNA expression, 600 ng of RNA was used as input for miRNA reverse transcription by the TaqMan miRNA Reverse Transcription Kit (Applied Biosystems, 4366597), and TaqMan miRNA Assays (Applied Biosystems, 4427975). The reverse transcription of the miRNA candidates and the housekeeping genes was conducted using the following cycle parameters: 16 °C for 30 min, followed by 60 cycles of 30 s at 20 °C, 30 s at 42 °C, and 50 °C for 2 s, and a final inactivation step of 5 min at 85 °C. All cDNA samples were applied in dilution of 1:10 to obtain results within the range of the standard. Commercially available TaqMan hydrolysis probes (Applied Biosystems) and SYBR Green I, with forward/reverse paired primers (KiCqStart SYBR Green Primers; Sigma, KSPQ12012), were used to analyze the expression of genes and miRNA candidates in a Light Cycler 480 II sequence detection system (Roche Diagnostics). The following thermocycler conditions were used: 50 °C for 2 min and 95 °C for 10 min, followed by 40 cycles for 30 s at 95 °C, 45 s at 60 °C, and 72 °C for 30 s. The level of gene and miRNA expression was normalized to the reference gene transcript *cyclophilin A* and *RNU6b* RNA, respectively. The normalized fold expression was obtained using the 2^−ΔΔCt^ method. The results were expressed as the normalized fold expression for each gene as compared to untreated (i.e., un‐induced) control cells. Replicates and positive and negative controls were included in all reactions. TaqMan hydrolysis assays and paired SYBR Green primer sequences are listed in Table S5, Supporting Information.

### Western Blotting

Protein analysis by western blot was performed on human preadipocytes and Wt and miR‐424(322)/503‐null MEFs following adipogenesis. Cells were washed with cold PBS and lysed with protein lysis buffer (Cell Signaling Technology, 9803). Protein concentrations were determined using a protein assay kit (Life Technologies, 22660/22663) and equal amounts of proteins were subjected to SDS‐PAGE and transferred to nitrocellulose membranes (Whatman, 10600001). Non‐specific binding was blocked by incubation with TBST (20 mm Tris‐Hcl pH7.4, 150 mm NaCl, 0.1% Tween‐20) plus 5% of non‐fat milk (Bio‐Rad, 170–6404). Membranes were incubated overnight at 4 °C with a 3% BSA (Fisher BioReagents, BP9703‐100)‐TBST solution containing the primary antibody. Thereafter, membranes were incubated for 1 h with secondary HRP‐conjugated antibodies at room temperature (Jackson Immunoresearch, 115‐035‐003 and 111‐035‐003). Signal was detected with a western blot HRP substrate (Millipore, WBLUF0500). The antibodies used in this study include anti‐SNCG (Sigma Aldrich, HPA014404), Anti‐TNFAIP8 (Sigma Aldrich, HPA057089), anti‐UNG (GeneTex, GTX113860), anti‐CHRDL1 (GeneTex, GTX49225), anti‐CEBP‐*α* (Santacruz, sc‐166258) anti‐AdipoQ (Life Technologies, PA1‐24411), anti‐PPAR*γ* (Santacruz, sc‐7273),anti‐FABP4 (R&D Systems, MAB3150) and anti‐Actin (Sigma Aldrich, A5441).

### DNA Methylation

The QIAamp DNA Blood Mini Kit (Qiagen, 51106) was used to extract DNA from the following: human primary preadipocytes; adipocytes after 14 days of transient differentiation; and subcutaneous adipose tissue from 5 morbid obese women before and ≈2 years after bariatric Roux‐en‐Y gastric bypass. Extracted DNA (500 ng per sample) was subjected to denaturation and bisulfite conversion using the EZ DNA Methylation‐Gold kit (Zymo Research, D5005), following manufacturer's instructions. PCR‐based amplification of specific CpG sites within the miR‐424(322)/503 cluster genomic region was achieved using the following primer sequences: FWD primer 5′‐GtAGtAATTtATGTTTTGAAGTGT‐3′, REV primer 5′‐CCAaCCTAaCCAaaAATACT‐3′. For PCR, the GC‐RICH PCR system (Roche) was applied. A total of 40 ng of bisulfite converted DNA was used in each case for PCR amplification. PCR conditions were 94 °C for 3 min and 45 cycles of 94 °C 30 s, 55 °C 45 s and 72 °C 1 min and 30 s, followed by 7 min at 72 °C for final elongation. PCR products were purified (Qiagen, 28104) and inserted into pGEM‐T Easy vector (Promega, A1360). The ligation product was transformed into DH5*α* competent cells (Invitrogen, 18265017), and at least 10 colonies per sample were sequenced (30 colonies per condition). For Sanger sequencing, the primers were: T7 (5′‐TAATACGACTCACTATAGGG‐3′) and SP6 (5′‐ATTTAGGTGACACTATAGAA‐3′). The methylation percentage of each analyzed CpG site was then calculated. All procedures were performed in triplicate. Transcription factor binding sites with CpG sites sensitive to DNA methylation and located within the miR‐424(322)/503 loci in Figure S3D, Supporting Information was retrieved from Mulan^[^ ^94^ ^]^ and used to infer pathway analysis enrichment using Enrichr^[^ ^95^, ^96^, ^97^ ^]^ and barplots of selected gene sets were represented using ggplot2.

### Adipose Lipids

Lipidomes were performed at the Lipidomics Lab Regensburg of the Institute of Clinical Chemistry and Laboratory Medicine at the University Hospital of Regensburg. Prior to lipid extraction, fat samples were spiked with internal standards, and solvent of standards was removed by vacuum centrifugation. Then, samples of subcutaneous adipose tissue from 20 miR‐KO mice and Wt controls under NC (1:1, male/females) were homogenized with a Precellys 24 tissue homogenizer (Bertin Instruments). To do so, 25–75 mg were added to a cup with ceramic beads (*V* = 2 mL). Methanol/chloroform (2:1, v/v) was applied in order to resuspend the samples at a final concentration of 0.05 mg µL^−1^. The homogenizer operated at 5000 rpm, and included two cycles of 15 s, and a 60 s break interval between cycles. Homogenates containing a wet weight of 2 mg were extracted according to the protocol described by Bligh and Dyer,^[^ ^98^ ^]^ with a total chloroform volume of 2 mL. A volume of 1.1 mL (for QQQ; FIA‐MS/MS) and 0.5 mL (for FIA‐FTMS) of the separated chloroform phase was transferred by a pipetting robot (Tecan Genesis RSP 150) and vacuum dried. The residues were dissolved in 1.1 mL methanol/chloroform (3:1, v/v) with 7.5 mm ammonium acetate or 1.2 mL chloroform/methanol/2‐propanol (1:2:4 v/v/v) with 7.5 mm ammonium formate, respectively. Lipid species were annotated according to the proposal for shorthand notation of lipid structures that were derived from mass spectrometry.^[^ ^99^ ^]^ The analysis of lipids was performed by direct flow injection analysis (FIA), using either a triple quadrupole mass spectrometer (QQQ; FIA‐MS/MS) or a hybrid quadrupole‐Orbitrap high‐resolution mass spectrometer (FIA‐FTMS). Both instruments were equipped with a heated electrospray ionization source. FIA‐MS/MS (QQQ) was performed in positive ion mode using the analytical setup and strategy described previously.^[^ ^100^, ^101^ ^]^ The following neutral losses were applied: PE, 141 and PI, 277.^[^ ^102^ ^]^ PE‐based plasmalogens (PE P) were analyzed according to the principles described by Berry and colleagues.^[^ ^103^ ^]^ Sphingosine‐based Cer was analyzed using a fragment ion of *m/z* 264.^[^ ^101^ ^]^ Correction for isotopic overlap of lipid species was performed for all lipid classes. Detailed description of the FIA‐FTMS method was published recently.^[^ ^104^, ^105^ ^]^ TG and DG were recorded in positive ion mode as [M+NH_4_]^+^ in *m/z* range 500–1000 and a target resolution of 140 000 (at *m/z* 200). PC and SM were analyzed as [M+HCOO]^−^ in negative ion mode in *m/z* range 520–960 at the same resolution setting. Peak assignment was performed with the ALEX software^[^ ^106^ ^]^ and applied a mass accuracy of less than 3 ppm. Quantification was achieved by multiplication of the spiked‐in IS amount with the analyte‐to‐IS intensity ratio. The following lipid species were applied as internal standards: Cer 18:1;O2/14:0, Cer 18:1;O2/17:0, DG 14:0/14:0/0:0, DG 20:0/20:0/0:0, HexCer 18:1;O2/12:0, HexCer 18:1;O2/17:0, LPC 13:0/0:0, LPC 19:0/0:0, PC 14:0/14:0, PC 22:0/22:0, PE 14:0/14:0, PE 20:0/20:0, PI 17:0/17:0, SM 18:1;O2/12:0, TG 17:0/17:0/17:0, and TG 19:0/19:0/19:0. Lipid species were annotated according to the recently published proposal for shorthand notation.^[^ ^107^ ^]^ Extracted data were processed by self‐programmed Macros as described in.^[^ ^106^ ^]^ Lipidomic data have been made publicly available in Figshare (10.6084/m9.figshare.16803235).

### 3'UTR Cloning and Mutagenesis

The 3“UTR regions of CHRDL1, SNCG, TNFAIP8, and UNG were cloned downstream of the luciferase‐reporter in the pMIR‐REPORT vector (Life Technologies, AM5795M) by PCR from human genomic DNA using specific primers: SNCG‐F(PmeI)/‐R(HindIII): aagtttaaacAGCGTGGATGACCTGAAGAG/aaaagcttCAAGTTTTATTTGGAATCAT; CHRDL1‐F(PmeI)/‐R(HindIII): aagtttaaacGGACAACTAACGCAGAGTA/aaaagcttTGCACCTCAGAAGTCAAC; TNFAIP8‐F(PmeI)/‐R(HindIII): aagtttaaacGGACAACTAACGCAGAGTA/aaaagcttTGCACCTCAGAAGTCAAC; and UNG‐F(PmeI)/‐R(HindIII): aagtttaaacGCTGTATCCAACCACAAAC/aaaagcttACTTCTCTCGGTCTCTTATC. After amplicon visualization on an agarose gel, PCR products were excised and purified from the agarose (Qiagen, 28706). In parallel with 3 µG of the pMIR_REPORT vector, PCR products were digested with the restriction enzymes PmeI (NEB, R0560) and MluI (NEB, R3198). Digested PCR products were purified (Qiagen, 28104) and ligated with the opened plasmid using a 3:1 ratio (NEB, M0202). Ligations were subsequently transformed on DH5*α* (Invitrogen, 18265017) chemically competent bacteria. Plasmid DNA from up to ten bacterial clones for each UTR was extractes (Qiagen, 27106) and analyzed by Sanger‐seq to sequence validate cloned regions. Site directed mutagenesis of the miR‐424‐5p and miR‐503‐5p binding sites was performed using the QuickChange Site‐Directed Mutagenesis kit (Agilent, 200518). Primer sequences for mutagenesis of miR‐424/503 binding sites on the SNCG 3”UTR region: SNCG‐mutFWD/‐mutREV: GGTCCTTCTGACCCCACTTATTTTTTTGTGAATTTTTTTTTTAAATGATTCC/GGAATCATTTAAAAAAAAAATTCACAAAAAAATAAGTGGGGTCAGAAGGACC; TNFAIP8‐mutFWD/‐mutREV: TCAGAGAAGCGTGGGCAGTATTTTTTTGAAATGGTTCCTCATATAGT/ACTATATGAGGAACCATTTCAAAAAAATACTGCCCACGCTTCTCTGA; and UNG‐mutFWD/‐mutREV: CACAGCCCTAGTTTGGCGCCTttTttTCCTTGGTTTTGCCTGGTTAGACTT/AAGTCTAACCAGGCAAAACCAAGGAaaAaaAGGCGCCAAACTAGGGCTGTG.

### Luciferase Assays

To measure luciferase activity, phoenix cells were plated at 70% confluence in 96‐well black plates (Corning/Cultek, 3603/153603). 24 h later, cells were transfected with 50 ng of pMIR‐REPORT constructs containing the luc‐3'‐UTR sequences in combination with a Renilla normalization control and 100 nm artificial miRIDIAN hsa‐miR424‐5p Mimic (Dharmacon, C‐300717‐05‐0005), miRIDIAN hsa‐miR503‐5p Mimic (Dharmacon, C‐300841‐05‐0005), or a miRIDIAN Mimic negative control (Dharmacon, CN‐001000‐01‐05) using the TransIT‐X2 transfection reagent (Mirus, MIR6000). After 24 h, relative luciferase units (RLU) were measured using the Dual‐Glo Luciferase Assay System (Promega, E2940) on a GloMax‐Multi+Microplate Multimode Reader (Promega, SA3030).

### Circulating *γ*‐Synuclein

123 women, and 55 men aged between 20 and 70 year‐old (47 ± 10 years), including patients at the extremes of the weight continuum (57.3 obese participants), were enrolled. An independent sample of 19 morbidly obese patients (6 men) following bariatric surgery was analyzed before and after 1 year of surgery‐induced weight loss. The study protocol was approved by the Ethics Committee and the Committee for Clinical investigation (CEIC) of the “Hospital Universitari Dr. Josep Trueta de Girona.” Enzyme‐linked immunosorbent assays were used to measure *γ*‐Synuclein in human plasma (R&D, DY5745‐05) and in serum from miR‐KO and Wt mice under NC (LifeSpan Biosciences, Inc., LS‐F12685‐1). Intra and inter‐assay coefficients of variation were between 5 and 10%. Other biochemical assessments were performed as routine laboratory measures.

### Statistical Analysis

Where indicated, data are expressed as mean ± standard deviation (S.D.) or standard error of means (S.E.M.). Statistical analysis was performed using SPSS (IBM Analytics) or GraphPad Prism, version 8.0 (GraphPad Software), with a *P*‐value of less than 0.05 considered significant. Statistical significance was determined by the Fisher's exact *t*‐test in comparisons between two groups. Differences between paired samples were compared by paired Student's *t*‐test. One‐way ANOVA followed by Tukey's honestly significant difference post‐hoc test was used to compare multiple groups (3 and above) and determine which specific groups differed from each other. Statistical significance was assessed in time‐course experiments by adjusted ANOVA after applying Šidák's corrections to repeated measures. Spearman's test was employed in correlation analyses. Sample sizes (*n*) are mentioned in each figure legend.

## Conflict of Interest

The authors declare no conflict of interest.

## Author Contributions

R.R.‐B., J.L., and L.D.‐J. contributed equally to this work. R.R.‐B., J.M.S., F.J.O., and D.L.‐N. designed the research. R.R.‐B., J.M.S., F.J.O., and D.L.‐N. developed the project conception and prepared the manuscript. R.R.‐B., J.L., L.D.‐J., A.L., F.J.L., LC‐I, M.O., K.G., M.L.D., J.M.M.‐N., L.P.‐P., M.D.‐P., F.J.O., and D.L.‐N. conducted research (hands on conduct of the experiments and data collection). R.R.‐B., J.L., L.D.‐J., N.B., A.C.‐N., F.J.O., and D.L.‐N. analyzed data and performed statistical analysis. M.H., G.L., and V.M.O. ran untargeted lipidomics. M.A.‐R., W.R., and J.M.F.‐R. coordinated human samples, clinical information, written consents, and intellectual content collection. All authors contributed to data interpretation and revised the manuscript. J.M.F‐R., J.M.S., F.J.O., and D.L.‐N. are senior authors. F.J.O. and D.L.‐N. jointly directed this work.

## Supporting information

Supporting Information

## Data Availability

Complete RNA‐sequencing data that support the findings of this study have been deposited in the community‐endorsed repository Gene Expression Omnibus (GEO) (http://www.ncbi.nlm.nih.gov/geo/) with the accession code number GSE185819. Lipidomic data have been made publicly available in Figshare (10.6084/m9.figshare.16803235)
