# Supplementary material for: A microRNA Cluster Controls Fat Cell Differentiation and Adipose Tissue Expansion By Regulating SNCG
Source: Adv Sci (Weinh). 2021 Dec 13;9(4):2104759. doi: 10.1002/advs.202104759 (PMC8811811; doi:10.1002/advs.202104759)

ADVANCED SCIENCE
Open Access

Supporting Information

for *Adv. Sci.*, DOI: 10.1002/advs.202104759

A microRNA Cluster Controls Fat Cell Differentiation and Adipose Tissue Expansion By Regulating SNCG

Ruth Rodríguez-Barrueco, Jessica Latorre, Laura Devis-Jáuregui, Aina Lluch, Nuria Bonifaci, Francisco J. Llobet, Mireia Olivan, Laura Coll-Iglesias, Katja Gassner, Meredith L. Davis, José M. Moreno-Navarrete, Anna Castells-Nobau, Laura Plata-Peña, Miki Dalmau-Pastor, Marcus Höring, Gerhard Liebisch, Vesa M. Olkkonen, Maria Arnoriaga-Rodríguez, Wifredo Ricart, José M. Fernández-Real, José M. Silva,* Francisco J. Ortega,* and David Llobet-Navas**

SUPPORTING TABLES

Table S1. Characteristics of study subjects, including miRNA candidates and gene expression measures taken in fat samples of obese participants with or without impaired glucose tolerance, and in non-obese subjects with normal glucose tolerance.

	Non-obese with NGT	Obese with NGT	Obese with IGT	p-value[a]	p-value[b]	p-value[c]	p-value[d]
n (men/women)	10/24	5/26	2/18				
Age (years)	48 ± 11	43 ± 11	47 ± 11	0.140	0.142	0.937	0.328
BMI (kg / m^2)	24.2 ± 3.4	45.3 ± 8.2	43.7 ± 5.6	**<0.0001**	**<0.0001**	**<0.0001**	0.596
Fat mass (%)	31.5 ± 6.3	57.3 ± 10.7	57.4 ± 7.2	**<0.0001**	**<0.0001**	**<0.0001**	0.999
SBP (mmHg)	122.5 ± 14.3	132.5 ± 20.5	152.0 ± 21.2	**<0.0001**	0.112	**<0.0001**	**0.009**
DBP (mmHg)	73.1 ± 12.5	75.6 ± 11.4	86.9 ± 12	**0.005**	0.731	**0.003**	**0.031**
Fasting glucose (mg / dl)	89.0 ± 8.8	89.3 ± 9.6	137.8 ± 57.8	**<0.0001**	0.999	**<0.0001**	**<0.0001**
Hb1Ac (%)	5.3 ± 0.4	4.8 ± 0.3	6.1 ± 1.7	**0.002**	0.273	**0.048**	**0.001**
Cholesterol (mg / dl)	199.9 ± 39.8	196.7 ± 31.2	201.9 ± 35.3	0.867	0.931	0.978	0.863
LDL (mg / dl)	119.8 ± 34.6	118.5 ± 30.9	120.2 ± 32.7	0.983	0.987	0.999	0.985
HDL (mg / dl)	60.1 ± 19.3	64.9 ± 57.6	53.0 ± 11.6	0.555	0.866	0.785	0.523
Triglycerides (mg / dl)	97.5 ± 49.9	106.8 ± 66.1	152.5 ± 98.7	**0.016**	0.857	**0.016**	0.059
Gene/miRNA expression in SC fat							
LEP	0.484 ± 0.303	0.930 ± 0.341	0.773 ± 0.252	**<0.0001**	**<0.0001**	**0.006**	0.254
ADIPOQ	2.95 ± 1.52	8.37 ± 5.88	5.81 ± 2.40	**<0.0001**	**<0.0001**	0.116	0.195
IRS1	0.0160 ± 0.0102	0.0130 ± 0.0079	0.0102 ± 0.0032	0.102	0.361	0.104	0.588
GLUT4	0.0597 ± 0.0335	0.0395 ± 0.0189	0.0368 ± 0.0229	**0.001**	**0.006**	**0.003**	0.914
FASN	1.037 ± 0.998	0.124 ± 0.214	0.062 ± 0.058	**<0.0001**	**<0.0001**	**<0.0001**	0.954
miR-424-5p	0.2651 ± 0.2627	0.0807 ± 0.0894	0.0439 ± 0.0345	**<0.0001**	**<0.0001**	**<0.0001**	0.67
CHRDL1	0.139 ± 0.071	0.235 ± 0.093	0.251 ± 0.074	**<0.0001**	**<0.0001**	**<0.0001**	0.746
SNCG	0.194 ± 0.134	0.359 ± 0.135	0.417 ± 0.172	**<0.0001**	**<0.0001**	**<0.0001**	0.285
TNFAIP8	0.0087 ± 0.0044	0.0114 ± 0.0040	0.0136 ± 0.0061	**0.001**	0.056	**<0.0001**	0.172
UNG	0.0095 ± 0.0038	0.0128 ± 0.0046	0.0133 ± 0.0034	**<0.0001**	**0.002**	**0.001**	0.892
Gene/miRNA expression in OM fat							
LEP	0.193 ± 0.160	0.313 ± 0.241	0.261 ± 0.108	0.116	0.106	0.61	0.791
ADIPOQ	2.52 ± 1.29	6.23 ± 8.39	4.90 ± 2.33	**0.049**	**0.048**	0.362	0.776
IRS1	0.0128 ± 0.0069	0.0095 ± 0.0031	0.0104 ± 0.0056	0.068	0.066	0.262	0.846
GLUT4	0.0517 ± 0.0369	0.0208 ± 0.0099	0.0185 ± 0.0141	**<0.0001**	**<0.0001**	**<0.0001**	0.929
FASN	0.758 ± 0.675	0.087 ± 0.139	0.054 ± 0.045	**<0.0001**	**<0.0001**	**<0.0001**	0.957
miR-424-5p	0.0336 ± 0.0321	0.0219 ± 0.0210	0.0128 ± 0.0131	**0.007**	0.135	**0.005**	0.363
CHRDL1	0.105 ± 0.055	0.134 ± 0.051	0.152 ± 0.042	**0.002**	0.051	**0.001**	0.345
SNCG	0.111 ± 0.078	0.128 ± 0.098	0.144 ± 0.068	0.333	0.682	0.304	0.759
TNFAIP8	0.0113 ± 0.0050	0.0125 ± 0.0043	0.0146 ± 0.0039	**0.023**	0.545	**0.018**	0.172
UNG	0.0115 ± 0.0045	0.0119 ± 0.0039	0.0128 ± 0.0038	0.484	0.915	0.459	0.688

Values represent mean ± standard deviation (S.D.). N/IGT: normal/impaired glucose tolerance, BMI: body mass index, S/DBP: systolic/diastolic blood pressure, HbA1c: glycated haemoglobin, H/LDL: high/low density proteins, SC/OM: subcutaneous/omental, *LEP*: leptin, *ADIPOQ*: adiponectin, *IRS1*: insulin receptor substrate 1, *GLUT4*: solute carrier family 2 (facilitated glucose transporter), member 4, *FASN*: fatty acid synthase, *CHRDL1*: chordin like 1, *SNCG*: synuclein gamma, *TNFAIP8*: TNF alpha induced protein 8, *UNG*: uracil DNA glycosylase. [a] One-way ANOVA followed by Tukey's honestly significant difference (HSD) post-hoc test for non-obese controls vs. obese subjects [b] without or [c] with IGT, and [d] for obese participants with and without IGT. Obesity was set at BMI≥30 kg/m^2. IGT was defined as impaired fasting glucose (≥110 mg / dl), or HbA1c of ≥5.7%. P values of less than 0.05 (marked in **bold**) were considered significant.

Table S2. Spearman's rho tests and rank-based correlations between the expression of our miRNA candidates and study variables and target genes (n=85).

Correlations - Rho (p-value)	SC miR-424-5p	OM miR-424-5p
Age (years)	**0.270 (0.009)**	0.017 (0.872)
BMI (kg / m^2)	**-0.375 (<0.0001)**	**-0.249 (0.019)**
Fat mass (%)	**-0.377 (<0.0001)**	-0.136 (0.07)
SBP (mmHg)	-0.071 (0.574)	-0.06 (0.637)
DBP (mmHg)	-0.046 (0.714)	0.088 (0.486)
Glucose (mg / dl)	-0.124 (0.248)	-0.083 (0.449)
HbA1c (%)	0.126 (0.348)	-0.053 (0.698)
Cholesterol (mg / dl)	0.166 (0.128)	0.057 (0.61)
HDL (mg / dl)	0.075 (0.505)	0.21 (0.064)
LDL (mg / dl)	0.142 (0.212)	-0.043 (0.714)
Triglycerides (mg / dl)	-0.064 (0.562)	-0.023 (0.836)
LEP in SC / OM	-0.142 (0.24)	0.089 (0.531)
ADIPOQ in SC / OM	**-0.369 (0.004)**	-0.003 (0.983)
IRS1 in SC / OM	**0.269 (0.021)**	0.181 (0.12)
GLUT4 in SC / OM	**0.415 (<0.0001)**	0.049 (0.648)
FASN in SC / OM	**0.518 (<0.0001)**	0.096 (0.387)
CHRDL1 in SC / OM	**-0.344 (<0.0001)**	-0.018 (0.84)
SNCG in SC / OM	**-0.312 (0.002)**	-0.062 (0.567)
TNFAIP8 in SC / OM	**-0.395 (<0.0001)**	-0.029 (0.789)
UNG in SC / OM	-0.154 (0.143)	0.039 (0.719)
OM miR-424-5p	0.118 (0.277)	---

SC/OM: subcutaneous/omental adipose tissue, BMI: body mass index, S/DBP: systolic/diastolic blood pressure, HbA1c: glycated haemoglobin, H/LDL: high/low density proteins, *LEP*: leptin, *ADIPOQ*: adiponectin, *IRS1*: insulin receptor substrate 1, *GLUT4*: solute carrier family 2 (facilitated glucose transporter), member 4, *FASN*: fatty acid synthase, *CHRDL1*: chordin like 1, *SNCG*: synuclein gamma, *TNFAIP8*: TNF alpha induced protein 8, *UNG*: uracil DNA glycosylase. P values of less than 0.05 were considered significant and marked in **bold**.

Table S3. Characteristics and gene and miRNA expression levels in subcutaneous adipose of 23 morbid obese women before (Baseline) and ~2 y after surgery (Post-WL).

Parameters	Baseline	Post-WL	p-value[a]
Age (years)	48 ± 10	51 ± 10	**<0.0001**
BMI (kg / m^2)	43.4 ± 5.0	29.2 ± 5.7	**<0.0001**
Fat mass (%)	56.2 ± 7.5	39.9 ± 7.7	**<0.0001**
SBP (mmHg)	129.2 ± 13.8	129.0 ± 16.3	0.870
DBP (mmHg)	79.3 ± 10.2	74.0 ± 12.8	0.163
Glucose (mg / dl)	101.2 ± 36.8	87.4 ± 14.1	0.106
HbA1c (%)	5.8 ± 1.5	5.3 ± 0.3	0.205
Cholesterol (mg / dl)	179.4 ± 31.7	182.9 ± 53.5	0.942
HDL (mg / dl)	55.7 ± 13.5	74.1 ± 22.9	**<0.0001**
LDL (mg / dl)	102.3 ± 27.4	99.0 ± 29.8	0.336
Triglycerides (mg / dl)	107.9 ± 39.7	81.1 ± 29.8	**0.005**
LEP	0.9564 ± 0.5138	0.2931 ± 0.2025	**<0.0001**
ADIPOQ	2.87 ± 0.99	4.23 ± 1.89	**0.006**
IRS1	0.0103 ± 0.0041	0.0138 ± 0.0070	0.07
GLUT4	0.043 ± 0.034	0.0872 ± 0.0556	**0.006**
FASN	0.465 ± 0.354	1.59 ± 0.207	**<0.0001**
miR-424-5p	0.008 ± 0.006	0.021 ± 0.014	**<0.0001**
CHRDL1	0.205 ± 0.0653	0.1253 ± 0.0439	**<0.0001**
SNCG	0.3819 ± 0.16	0.1471 ± 0.0842	**<0.0001**
TNFAIP8	0.012 ± 0.0037	0.0052 ± 0.0026	**<0.0001**
UNG	0.0103 ± 0.0024	0.008 ± 0.002	**<0.0001**

Values represent mean ± standard deviation (S.D.). BMI: body mass index, S/DBP: systolic/diastolic blood pressure, HbA1c: glycated haemoglobin, H/LDL: high/low density proteins, SC/OM: subcutaneous/omental, *LEP*: leptin, *ADIPOQ*: adiponectin, *IRS1*: insulin receptor substrate 1, *GLUT4*: solute carrier family 2 (facilitated glucose transporter), member 4, *FASN*: fatty acid synthase, *CHRDL1*: chordin like 1, *SNCG*: synuclein gamma, *TNFAIP8*: TNF alpha induced protein 8, *UNG*: uracil DNA glycosylase. [a] Results post-weight loss *versus* baseline were compared by paired sample t-test. P values of less than 0.05 (marked in **bold**) were considered significant.

Table S4. Circulating γ-Synuclein in association with obesity and surgery-induced weight loss.

Cross-sectional study	NO women (n=53)	Ob women (n=70)	p-value[a]
Age (years)	50 ± 10	47 ± 11	0.118
BMI (kg / m^2)	24.3 ± 2.8	43 ± 6.8	**<0.0001**
Fat mass (%)	36 ± 5.8	51.9 ± 4	**<0.0001**
SBP (mmHg)	122.4 ± 16.5	135.3 ± 17	**<0.0001**
DBP (mmHg)	70.4 ± 9.8	76.3 ± 10.7	**0.002**
Glucose (mg / dl)	93.9 ± 11.1	96.4 ± 11.1	0.215
HbA1c (%)	5.5 ± 0.3	5.6 ± 0.3	0.06
Cholesterol (mg / dl)	204.8 ± 39.0	193.7 ± 42.1	0.137
HDL (mg / dl)	71.2 ± 18.3	52.2 ± 11.2	**<0.0001**
LDL (mg / dl)	121.8 ± 33.7	121.9 ± 42.5	0.99
Triglycerides (mg / dl)	77.5 ± 35.5	115.3 ± 53.1	**<0.0001**
γ-Synuclein (pg / ml)	84.3 [59.9-148.3]	150.7 [110.9-275.1]	**<0.0001**
Cross-sectional study	NO men (n=23)	Ob men (n=32)	p-value[b]
Age (years)	46 ± 11	44 ± 9	0.467
BMI (kg / m^2)	26.2 ± 2	45.4 ± 7.9	**<0.0001**
Fat mass (%)	25.9 ± 6.4	44.8 ± 4.9	**<0.0001**
SBP (mmHg)	128.1 ± 15.6	142.7 ± 20.9	**0.007**
DBP (mmHg)	75.9 ± 12.5	79.9 ± 10.8	0.209
Glucose (mg / dl)	95.0 ± 14.7	96.0 ± 11.9	0.797
HbA1c (%)	5.4 ± 0.2	5.6 ± 0.3	**0.001**
Cholesterol (mg / dl)	204.0 ± 42.1	188.3 ± 31.5	0.120
HDL (mg / dl)	54.3 ± 13.7	45.7 ± 13.2	**0.022**
LDL (mg / dl)	126.2 ± 34.7	117.2 ± 26.9	0.291
Triglycerides (mg / dl)	117.8 ± 73.7	128.9 ± 72	0.579
γ-Synuclein (pg / ml)	59.1 [46.3-155.5]	158.2 [91.4-316.3]	**0.018**

Longitudinal study	Baseline	Post-weight loss	p-value[c]
Age (years)	40 ± 10	42 ± 10	**<0.0001**
BMI (kg / m^2)	46.2 ± 6.7	29.1 ± 5.2	**<0.0001**
Glucose (mg / dl)	97.7 ± 23.4	86.9 ± 5.7	0.067
HbA1c (%)	5.7 ± 0.6	5.2 ± 0.3	**<0.0001**
Cholesterol (mg / dl)	179.5 ± 27.1	164.1 ± 30.8	**0.02**
HDL (mg / dl)	45.3 ± 8.1	59.6 ± 12.1	**<0.0001**
LDL (mg / dl)	113.3 ± 22.8	90.8 ± 30	**0.001**
Triglycerides (mg / dl)	104.6 ± 58	68.3 ± 20.2	**0.002**
Ultrasensitive CRP (mg / dl)	7.0 ± 9.8	1.5 ± 2.7	**0.004**
Cortisol (mcg / dl)	10.1 ± 3.5	12.3 ± 4.9	**0.039**
γ-Synuclein (pg / ml)	277.8 [128.8-455.8]	98.1 [70.1-215.2]	**<0.0001**

Values represent mean ± standard deviation (SD), or median and inter-quartile range (γ-synuclein). BMI: body mass index, S/DBP: systolic/diastolic blood pressure, HbA1c: glycated haemoglobin, H/LDL: high/low density proteins, CRP: C reactive protein. Obesity was set at BMI ≥ 30 kg/m^2. [a,b] Student's *t*-test was performed for non-obese (NO) *versus* obese (Ob) subjects in cross-sectional comparisons, and [c] paired sample *t*-test was applied to longitudinal changes following weight-loss in obese subjects (n=19; 6 men) after ~1 y of surgery. P values of less than 0.05 (marked in **bold**) were considered significant.

Table S5. Paired SYBR Green primers and TaqMan assays used during this research.

**Human genes**	**Forward SYBR Green primers**	**Reverse SYBR Green primers**
ACACB	CTGAGTCACGTGCATATC	ACAAGTAGGCCTTGACAG
ACSL1	TGAGTGGGTGATTATTGAAC	GTTGACTATGTACGTGATGG
ALDH6A1	AGCCAAATATGACCTGTTAC	CATCCAATGTTTCTGTCTCC
ANKRD9	GAGATCAAGAGAGGTATGGG	ATGTGGGTATATCACAGTCC
CADM3	CTTTCCTCAACAAGAGTGAC	GTCATTAACATTGAGGGTGTAG
CHRDL1	CTATTCGAAAGGGCATTCTC	CCTTGACTAAATCTTCAAGCTC
FASN	CAATACAGATGGCTTCAAGG	GATGTATTCAAATGACTCAGGG
G0S2	TGCCACTAAGGTCATTCC	TTCACCATCTTCCCCTTG
GLUL	GTGAAGACTTTGGAGTGATAG	GATGTACTTCAGACCATTCTC
GPAM	GGATATTGGGGTTTTCAAGG	TACCTACAAGGAACTCATCTC
GPER1	AGGTACCCAGAGAGTGAG	AGTGGGAAGAACAGATGC
KNCK3	ATTCTCATAGCAGGTAGGAC	CAGAGGCTAAAAATCACTCG
LDHD	CTCACAGGAAGCATTGTC	TGTTCTGCAAAAGCCTTG
LIPE	CTATGCTGGTGCAAAGAC	CTCCAGGAAGGAGTTGAG
MLYCD	CTCTCAGAATGGTTTTCCTC	CCTCACTGATTTTCTGAAGC
NAT8L	ACAAAACGGTAGTTTGTACC	GAAATGACATCCAATCAGGG
NGFR	AACCTCATCCCTGTCTATTG	CCTCTTGAAGGCTATGTAGG
PDE3B	AAATTCTGGAGGTGGAAATG	ATACTCCGTAGAGAGGAAATG
PPP1R1A	GTCATCCCCAGAGATAGATG	CATCTGGAGCTCTTTCATTG
SNCG	ATGGATGTCTTCAAGAAGGG	CTCCCACATACATGACCC
TMEM135	AAGTCCATCCCTCATAACTG	ATCAAGTACAGAGGAGCATAG
TNFAIP8	GGGAGGTTTTGATTTTAGTGG	CACTTCCTTGGATTCTTCTG
UNG	TCATGGACCTAATCAAGCTC	TAAATGTTCTCCAAACTGGG
**Mice genes**	**Forward SYBR Green primers**	**Reverse SYBR Green primers**
Acacb	GCATGAAGGACATGTATGAG	AGGGATGTAGATGAGAATGG
Chrdl1	AACAGAGAAGCAAGACATTC	GCTTGCTGGGAATCTATAAC
G0s2	AGCTGAGGGAAGAAGAAC	TATAGCTTCACTAGCTTCCC
Glul	CAAGTGTGTGGAAGAGTTAC	TGAAAACTTCACATAGCACC
Pde3b	AGGATTCTCAGTCAGGTTATG	GTTGTCAAATACCAAACAGC
Ppp1r1a	AAAAGTCTGCAGAATCCAAC	GTCCATGAATTTCCACACTG
Sncg	ATGAAGAGGCCAAGAGTG	GCTAGGGACAGAGAACTTG
Tnfaip8	CTCCGATGGCTACAGATG	ATTTGGATACCATTTTGCCC
Ung	AATGGGATTTGTTGCTGAAG	TTGATTAGGTCCGTGATAGG
Pdgfra	CTAGTTCCTGCATCCATTTTG	ATATTTGAGACATTGCTGGC
Pdgfrb	ATCTCTGTGATCGAGAATGG	AGTAGACAAAACTAACTCGC
mH19X	CCCCAAATCTAGGCTCCTTTGT	ATCAGGACTGACTCATTTGGTGG

**Human genes**	**Ref. # TaqMan assays**	**Mice genes**	**Ref. # TaqMan assays**
ADIPOQ	Hs00605917_m1	*Prdm16*	Mm00712556_m1
CCND3	Hs00236949_m1	*Scd1*	Mm00772290_m1
CDK4	Hs00364847_m1	*Srebf1*	Mm00550338_m1
ELOVL6	Hs00907564_m1	*Tnf*	Mm99999068_m1
FASN	Hs01005622_m1	*Ucp1*	Mm01244861_m1
GLUT4	Hs00168966_m1	*Glut4*	Mm00436615_m1
IGF1R	Hs00609566_m1	*Irs1*	Mm01278327_m1
IL6	Hs00985639_m1	*Plin1*	Mm00558672_m1
IRS1	Hs00178563_m1	*Pparg*	Mm00440940_m1
LEP	Hs00174877_m1	*Ppargc1a*	Mm01208835_m1
PNPLA2	Hs00982042_m1	*Ppia*	Mm02342430_g1
PPIA	Hs99999904_m1	**Target miRNAs**	**Ref. # TaqMan assays**
TGFB1	Hs00998133_m1	hsa-miR-424-5p	000604
**Mice genes**	**Ref. # TaqMan assays**	hsa-miR-503-5p	001048
Acsl1	Mm00484217_m1	mmu-miR-322-5p	001076
Adipoq	Mm00456425_m1	mmu-miR-503-5p	002456
Adrb3	Mm02601819_g1	U6 snRNA	001973
Fabp4	Mm00445880_m1		
Fasn	Mm00772290_m1		

SUPPLEMENTAL FIGURE LEGENDS

Figure S1. (**A**) Visceral deposits of white adipose tissue in dissected wild-type (Wt) and miR-424(322)/503 knockout (miR-KO) female mice. (**B**) Representative images of dual-energy X-ray absorptiometry (DEXA) in Wt and miR-KO males and females under normal chow (NC). Right bar graph shows quantification of bone mineral density and content (n=7/genotype/sex). (**C**) Body length (cm) in Wt and miR-KO male and female mice of 3 months of age. (**D**) Biochemical characterization of circulating cholesterols, triglycerides and free fatty acids (fFA), and (**E**) intraperitoneal glucose tolerance test (GTT) performed in NC/high-fat diet (HFD)-fed adult Wt/miR-KO males and females (n=7 mice/genotype/sex). The mice were fasted for 12 h, and the baseline blood glucose was measured at 0 min. Then, 1 gram of glucose/kg body weight was injected. Glucose levels were tested at regular intervals of 15, 30, 60, and 120 min. Dashed lines depict results in miR-KO mice. Quantification of the area under the curve (AUC) is shown in **Figure 1H**. Data are presented as mean ± S.E.M. (**F**) Representative flow cytometry plots illustrating the sequential process used to analyse the subpopulation of resident epididymal (eWAT) and subcutaneous (scWAT) adipose-derived stem cells (ASCs) and pre-adipocytes (see in **Figure 1I**). (**G**) Expression of adipose Zinc finger protein 423 (*Zfp423*) and Kruppel Like Factor 14 (*Klf14*) as marker genes of adipocyte commitment/ preadipocyte performance (n=4 mice/genotype/sex). n.u. stands for "normalized units". (**H**) Estimation of fat cell-numbers (as volume of fat deposit/average adipocyte's volume) and (**I**) histone H3 phosphorylation on serine-10 in Wt and miR-KO female mice (n=4 mice/genotype). P-values were calculated using Student's t-test. *p<0.05; **p<0.01; ***p<0.001. NS, not significant.

Figure S2. (**A** and **B**) Representative images of haematoxylin/eosin-stained histological sections of epididymal (eWAT) and subcutaneous (scWAT) adipose tissue from 5 mo-old Wt and miR-KO male mice maintained on NC and HFD, respectively (Scale bars, 50 μm). Dot plots representing area of adipocytes and Gaussian fit histograms depicting adipocyte area distribution are also shown. 500 adipocytes were measured per mouse (n=3/genotype/sex, thus 1500 adipocytes/genotype/sex). Females are depicted in **Figure 1J-K**. (**C**) Expression of adipogenic markers by real time-PCR in Wt and miR-KO male and female mice under NC (n=6/genotype/sex). P-values were calculated using Student's t-test. *p<0.05; **p<0.01; ***p<0.001; NS, not significant. Lipidomics of the scWAT of miR-KO and Wt male and female mice (n=5 per group) summarized a total of 187 different lipid species. Volcano plots show relative variations for specific lipids in both (**D**) female and (**E**) male mice. Node shape: circle, glycerolipids and glycerophospholipids (including DG, PE, PEP, PC, PI, and LPC); square, sphingolipids (including Cer, HexCer, and SM); triangle, triglycerides (TG). Edge colour for different lipid family. Each lipid was analysed individually, without assuming a consistent S.D. Significant variations were set at fold-changes >[1.5] and Student's *t*-test of P = 0.05 (red lines). Scatter plots at the right hand show variations in adipose TG according to the length and saturation stats of fatty acids.

Figure S3. (**A**) Target gene overlap between human/mouse hsa-miR-424/mmu-miR-322 and hsa-miR-503/mmu-miR-503. (**B**) Microarray expression measures of the miR-424 and the miR-503 (amongst others) in primary human obese/lean preadipocytes while differentiating into mature adipocytes (adapted from [1]). (**C**) Analysis of DNA methylation in human preadipocytes and lipid-containing mature adipocytes (>10 alleles per condition and biological replicate). (**D**) Pathway enrichment analysis of transcription factors binding sites sensitive to DNA methylation and located within the miR-424(322)/503 loci. Transcription factor binding sites with CpG sites were retrieved from Mulan [2] and used to infer pathway analysis enrichment using Enrichr [3–5].

Figure S4. (**A**) Schematic illustration of the pTRIPz doxycycline inducible system. DOX=doxycycline, TRE=tetracycline response element, rtTA3=reverse tetracycline/ doxycycline-regulated transactivator gene. (**B**) Expression of genes related to adipogenesis in undifferentiated mouse embryonic fibroblasts (MEFs) isolated from miR-KO (red) and Wt (green) fat, the later taken as reference (100%). Gene expression analysis in (**C**) undifferentiated adipocyte precursor cells maintained in human preadipocytes media (without additional hormones); and (**D**) during the first (0-7d), (**E**) during the last (7-14d), and for the last (**F**) 96 or (**G**) 48 hours of terminal differentiation. Expression patterns in pT-miR-424/503 adipocytes stimulated with Dox during the 14-day differentiation course are provided in **Figure 2L**. (**H**) Expression of adipocyte commitment marker genes in 3T3-L1 adipocytes challenged for 96 h with synthetic miRIDIAN-Mimic of mmu-miR-322-5p. (**I**) Expression of adipocyte marker genes and area occupied by vacuoles in ASC52telo adipocytes challenged with a synthetic miRIDIAN Hairpin Inhibitors directed against human miR-424 during the adipocyte course. Data are presented as mean ± S.E.M (n≥4). P-values were calculated using Student's t-test. *p<0.05; **p<0.01; ***p<0.001. NS, not significant. Proliferation of (**J**) 3T3-L1 and (**K**) ASC52telo adipocyte precursors cells respectively challenged with mimic mouse miR-322 and antagomiRs against human miR-424 show significant variations in cell growth. Statistical significance was assessed by adjusted ANOVA after applying Šidák's correction to repeated measures (n=3 biological replicates/cell/treatment/time).

Figure S5. (**A**) Scatter dots show the association of subcutaneous (SC; *p*=0.009) and omental (OM; ns) miR-424 with age in subjects depicted according to their weight and glucose tolerance (i.e., normal weight (NW) subjects with normal glucose tolerance (NGT) in green, and obese (OB) participants with impaired (IGT) and normal glucose tolerance in red and orange, respectively). Spearman's *p*-values and *r* correlation coefficients show the statistical dependence between these variables, if any. (**B**) Bean plots show cross-sectional variations in men (n=17) and women under (n=43) and over (n=25) 50-years old. Statistical significance for differences between groups was determined by One-way ANOVA followed by Tukey's honestly significant difference (HSD) post hoc tests). (**C**) Analysis of DNA methylation in human SC adipose tissue before/after bariatric surgery and massive weight loss (>10 alleles per time-point and subject; n=5 paired-samplers. See also in **Figure 3I**).

Figure S6. (**A**) RNA-seq was performed on Wt and miR-KO MEFs differentiated into mature adipocytes (MA). Upper left, principal component analysis (PCA) showing sample distribution. Upper right and down, gene set enrichment analysis (GSEA) was used to uncover enriched gene annotations from the molecular signatures database (MSigDB) in miR-KO adipocytes. Red and blue circles (nodes) represent different pathways exhibiting positive or negative enrichment, respectively (FDR q_val<0.05). Arrows interconnect annotations presenting common genes in the leading edge subset for each annotation. (**B**) Deep transcriptome sequencing and GSEA were also conducted on undifferentiated MEFs. MA plot illustrates the distribution of DEGs (upper right). (**C**) The relevance of the regulatory cluster miR-424(322)/503 on mouse and human adipocyte commitment reverberates through the modulation of hundreds of genes, many of them (in dark blue) defined as potential miR-424(322)/503 target genes, according to TargetScan (TS). The 24-gene set common signature in engineered and miR-KO human and mouse MA is individually labelled and highlighted in red.

Figure S7. The 24 target genes highlighted in complementary models of gain and loss-of-function were assessed by real time-PCR in human adipocytes after (**A**) 14 days (14d) of Dox-induced expression of the cluster miR-424(322)/503 (pT-miR-424/503) versus control (pT-Control); (**B**) engineered adipocytes expressing the miRNA cluster for 9 days after complete differentiation (9d); and (**C**) acute treatments of 48 h (48h). Each connecting arrow indicates the number of significant genes found in the first plot and the number of significant genes found in the following filtering plot. Inhibition of adipocyte differentiation after treatment with recombinant transforming growth factor β (TGFBβ) for 14 days is characterized by a decrease in the expression of such shortlisted miR-424(322)/503 target genes in both human (**D**) preadipocytes and (**E**) during the course of differentiation towards adipocytes. *PKDCC* did not show amplification data in any of the experimental settings, so it is excluded from plotting. Western blots (n=2) and measures of gene expression show changes in SNCG related to the modulation of miR-424(322) in adipocytes derived (**F** and **G**) from mouse 3T3-L1 or (**H**) from human ASC52telo cells. Plotted data are presented as mean ± S.E.M (n≥4). Unpaired Student's *t*-test was used for comparison between two groups. *p<0.05; **p<0.01; ***p<0.001. NS, not significant.

Figure S8. Expression patterns of mouse miR-322 and miR-503 in (**A**) Dgcr8-deficient brown adipocytes (Kim *et al.* [6]), (**B**) differentiated brown mature adipocytes when compared to preadipocytes (Chen *et al.* [7]), and (**C**) brown adipose tissue (BAT) of mice responding to cold exposure (Trajkovski *et al.* [8]). Also expression of *mH19X* in mice challenged with (**D**) an HFD-regime or (**E**) cold exposure (Schmidt *et al.* [9]) is shown, depicting no significant variations in BAT. (**F**) Expression of genes related to the performance/activity of BAT in wild-type (Wt) and miR-424(322)/503-null (miR-KO) male and female mice under regular laboratory conditions. Expression of gene markers for fat cell performance in Wt and miR-KO MEFs-derived adipocytes responding to recombinant TGFβ in (**G**) treatments of 48 h and (**H**) during adipogenesis (8 d). Data are presented as mean ± S.E.M (n=4 biological replicates/group). Unpaired

Student's *t*-test was used for comparison between two groups (i.e., miR-KO vs Wt males or females; males vs females; treated vs control). *p<0.05; **p<0.01; ***p<0.001. NS, not significant.

SUPPORTING REFERENCES

[1] F. J. Ortega, J. M. Moreno-Navarrete, G. Pardo, M. Sabater, M. Hummel, A. Ferrer, J. I. Rodriguez-Hermosa, B. Ruiz, W. Ricart, B. Peral, J. M. Fernández-Real, *PLoS One* **2010**, *5*, DOI 10.1371/journal.pone.0009022.

[2] I. Ovcharenko, G. G. Loots, B. M. Giardine, M. Hou, J. Ma, R. C. Hardison, L. Stubbs, W. Miller, *Genome Res.* **2005**, *15*, 184.

[3] Z. Xie, A. Bailey, M. V Kuleshov, D. J. B. Clarke, J. E. Evangelista, S. L. Jenkins, A. Lachmann, M. L. Wojciechowicz, E. Kropiwnicki, K. M. Jagodnik, M. Jeon, A. Ma'ayan, *Curr. Protoc.* **2021**, *1*, e90.

[4] M. V Kuleshov, M. R. Jones, A. D. Rouillard, N. F. Fernandez, Q. Duan, Z. Wang, S. Koplev, S. L. Jenkins, K. M. Jagodnik, A. Lachmann, M. G. McDermott, C. D. Monteiro, G. W. Gundersen, A. Ma'ayan, *Nucleic Acids Res.* **2016**, *44*, W90.

[5] E. Y. Chen, C. M. Tan, Y. Kou, Q. Duan, Z. Wang, G. V. Meirelles, N. R. Clark, A. Ma'ayan, *BMC Bioinformatics* **2013**, *14*, 128.

[6] H.-J. Kim, H. Cho, R. Alexander, H. C. Patterson, M. Gu, K. A. Lo, D. Xu, V. J. Goh, L. N. Nguyen, X. Chai, C. X. Huang, J.-P. Kovalik, S. Ghosh, M. Trajkovski, D. L. Silver, H. Lodish, L. Sun, *Diabetes* **2014**, *63*, 4045.

[7] Y. Chen, F. Siegel, S. Kipschull, B. Haas, H. Fröhlich, G. Meister, A. Pfeifer, *Nat. Commun.* **2013**, *4*, 1769.

[8] M. Trajkovski, K. Ahmed, C. C. Esau, M. Stoffel, *Nat. Cell Biol.* **2012**, *14*, 1330.

[9] E. Schmidt, I. Dhaouadi, I. Gaziano, M. Oliverio, P. Klemm, M. Awazawa, G. Mitterer, E. Fernandez-Rebollo, M. Pradas-Juni, W. Wagner, P. Hammerschmidt, R. Loureiro, C. Kiefer, N. R. Hansmeier, S. Khani, M. Bergami, M. Heine, E. Ntini, P. Frommolt, P. Zentis, U. A. Ørom, J. Heeren, M. Blüher, M. Bilban, J. W. Kornfeld, *Nat. Commun.* **2018**, DOI 10.1038/s41467-018-05933-8.

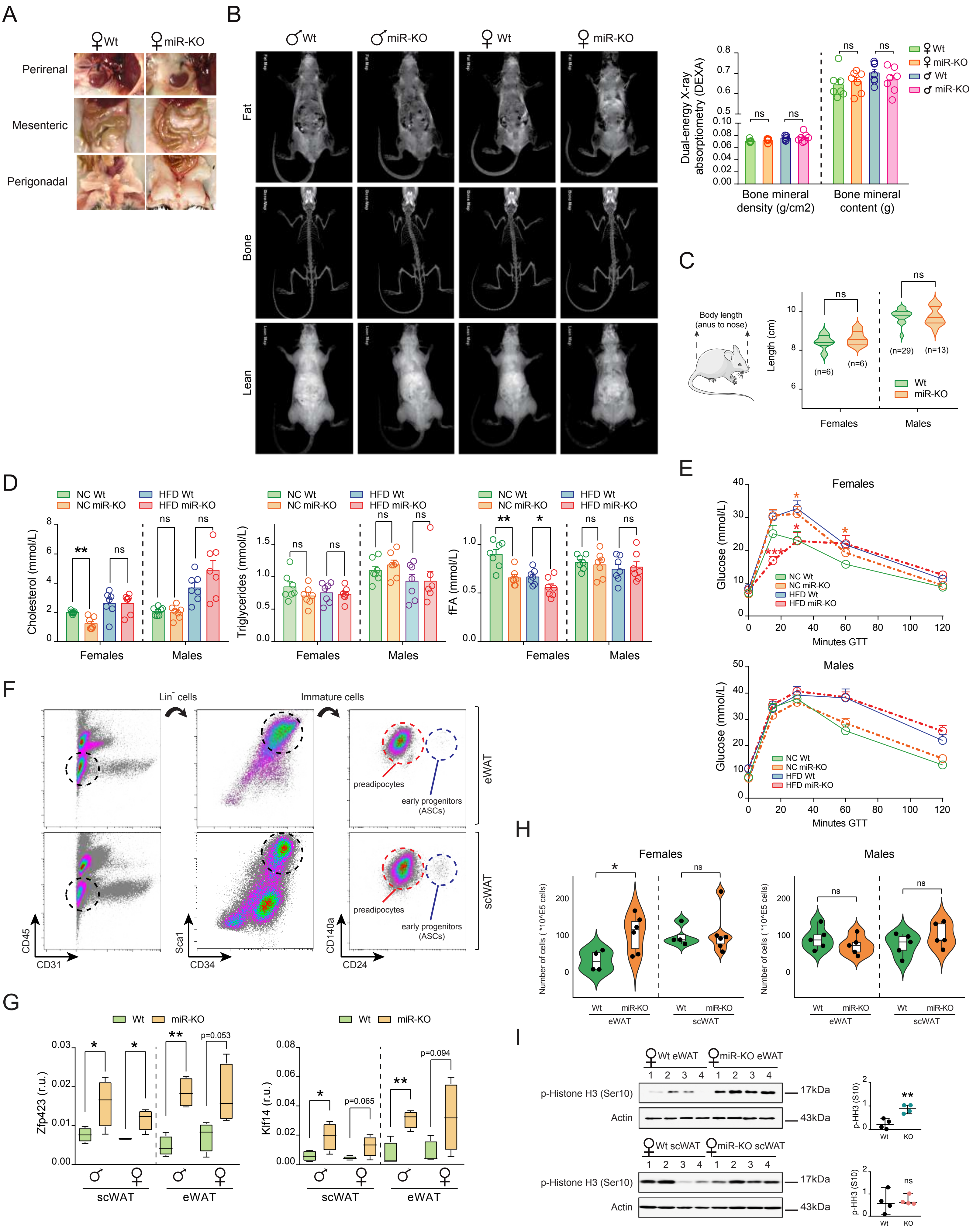

A
♀Wt
♀miR-KO
Perirenal
Mesenteric
Perigonadal
B
♂Wt
♂miR-KO
♀Wt
♀miR-KO
Fat
Bone
Lean
Dual-energy X-ray absorptiometry (DEXA)
Bone mineral density (g/cm2)
Bone mineral content (g)
ns
C
Body length (anus to nose)
Length (cm)
(n=6)
(n=29)
(n=13)
Wt
miR-KO
Females
Males
D
NC Wt
NC miR-KO
HFD Wt
HFD miR-KO
Cholesterol (mmol/L)
Triglycerides (mmol/L)
fFA (mmol/L)
E
Females
Males
Glucose (mmol/L)
Minutes GTT
F
Lin⁻ cells
Immature cells
preadipocytes
early progenitors (ASCs)
eWAT
scWAT
CD45
CD31
Sca1
CD34
CD140a
CD24
G
Zfp423 (r.u.)
Klf14 (r.u.)
p=0.053
p=0.065
p=0.094
H
Number of cells (*10^E5 cells)
I
♀Wt eWAT
♀miR-KO eWAT
♀Wt scWAT
♀miR-KO scWAT
p-Histone H3 (Ser10)
Actin
17kDa
43kDa
p-HH3 (S10)
Wt
KO

Suppl Figure-2 Rodriguez-Barrueco R

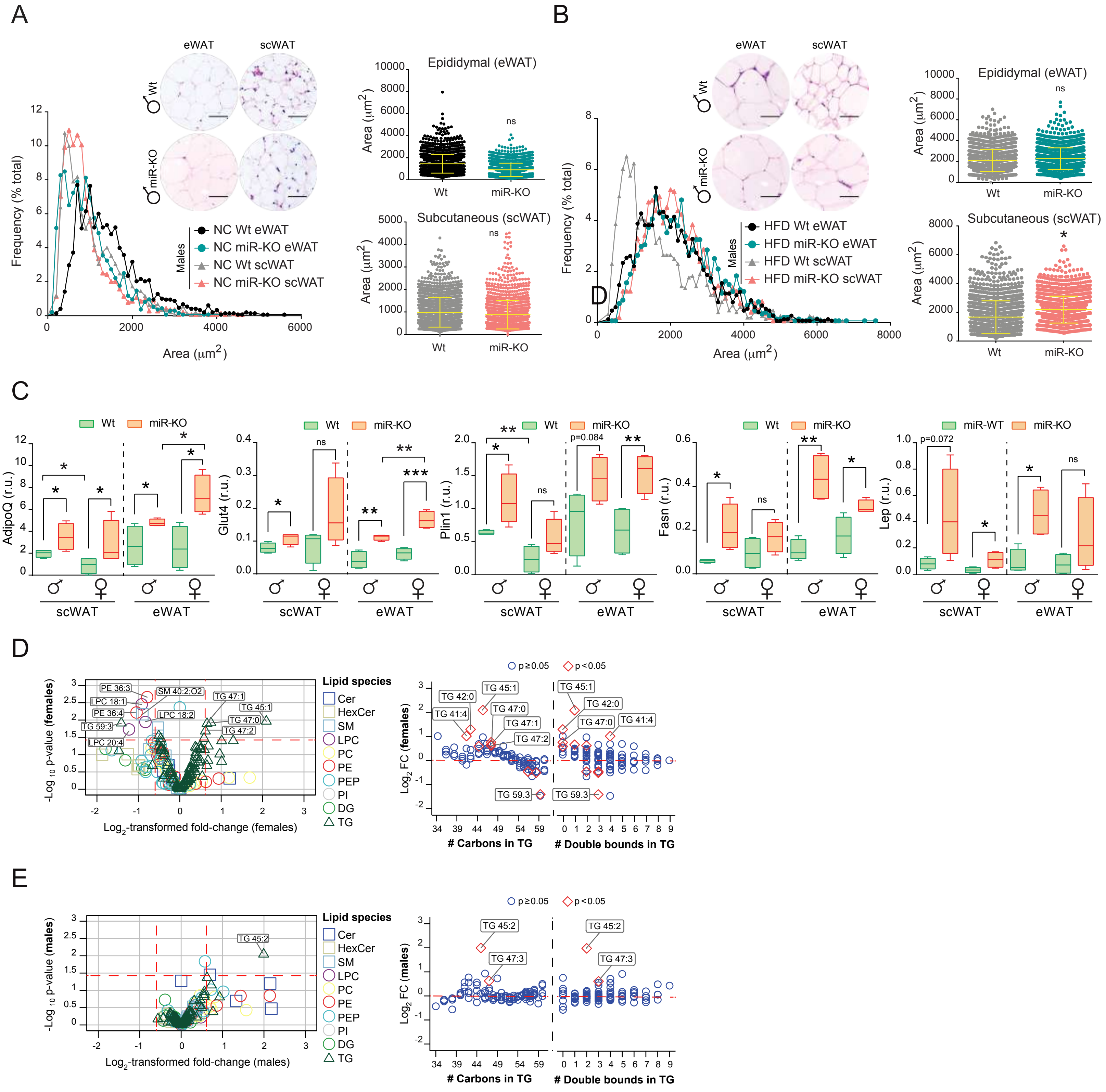

Suppl Figure-3 Rodriguez-Barrueco R

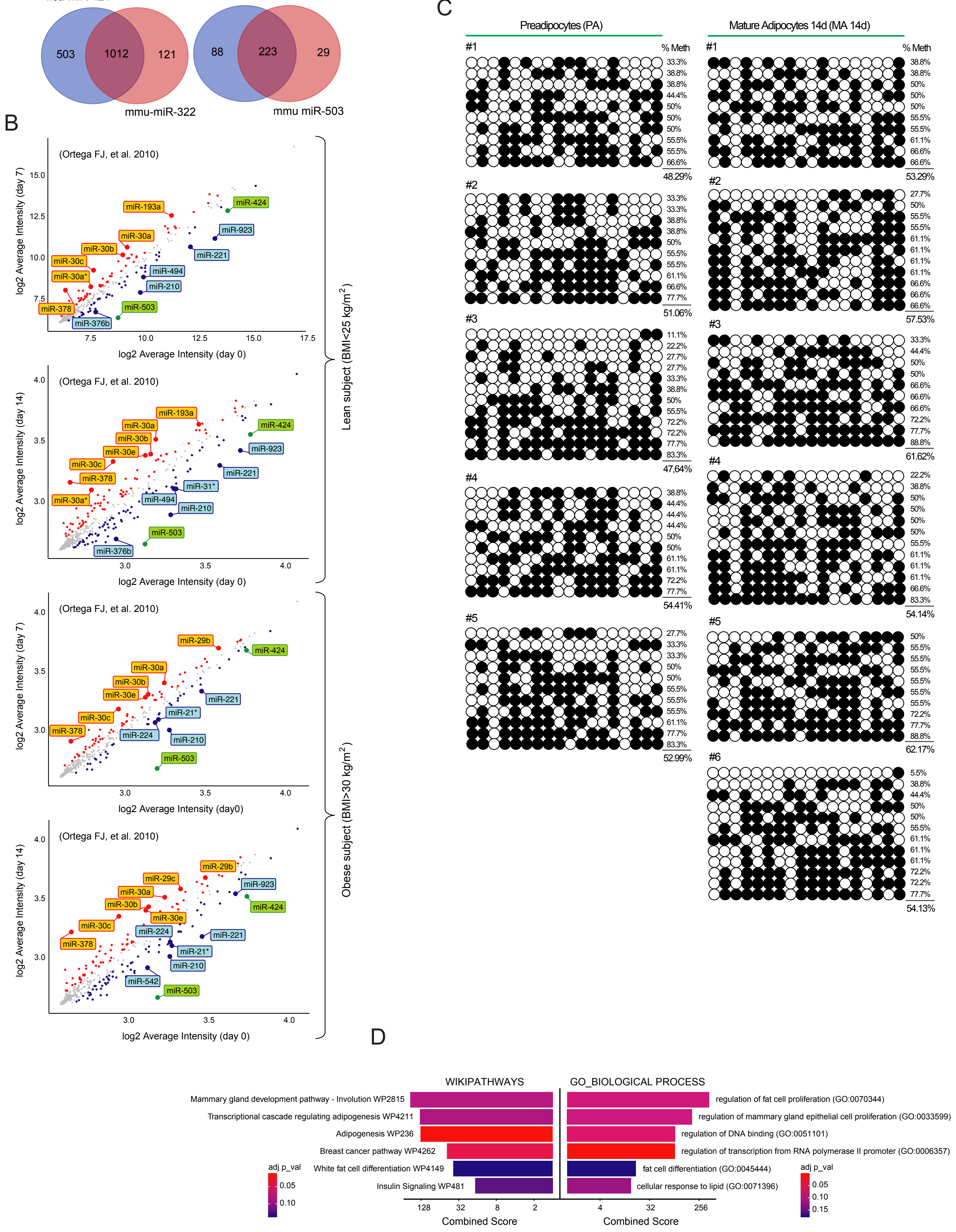

Suppl Figure-4 Rodriguez-Barrueco R

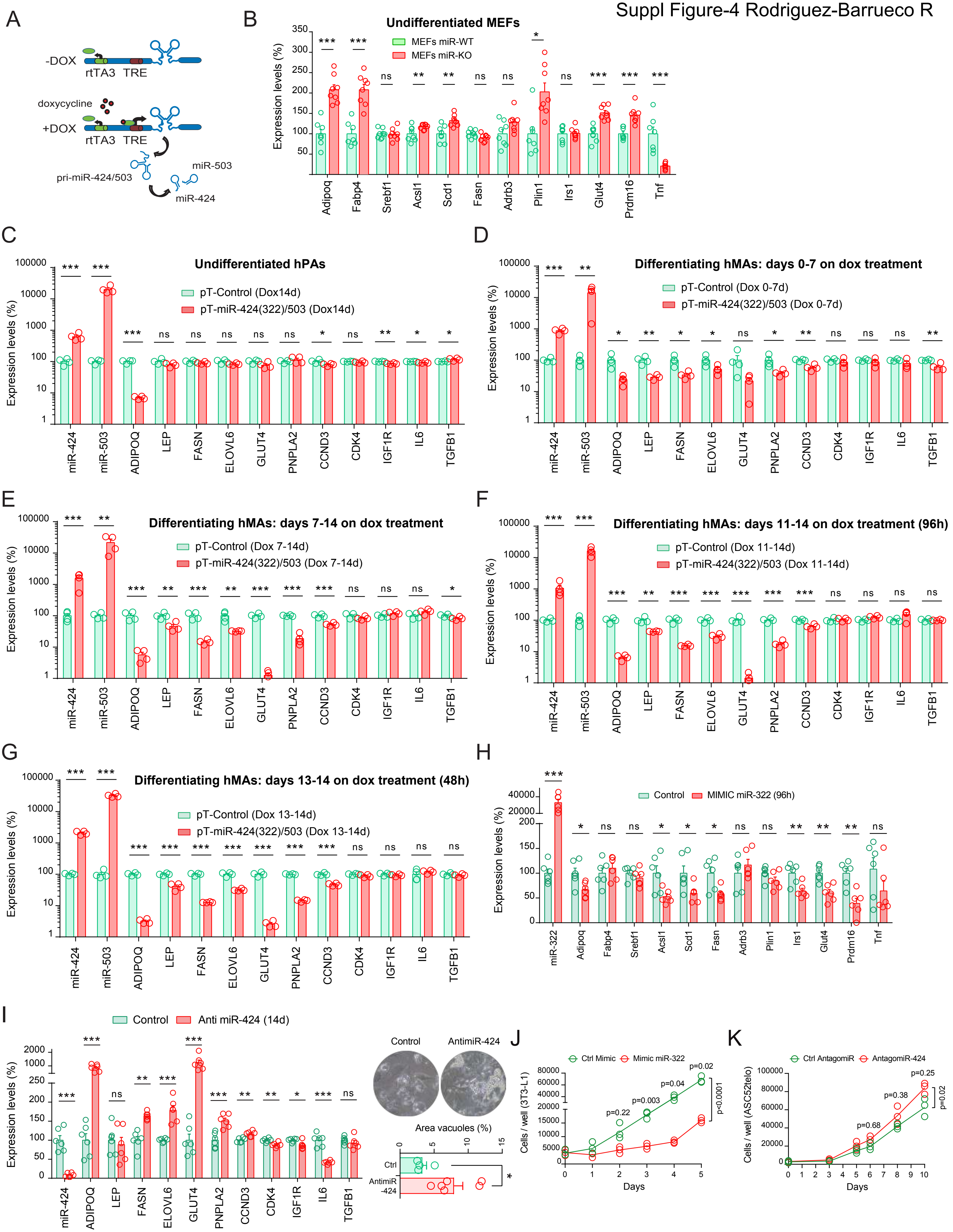

Suppl Figure-5 Rodriguez-Barrueco R

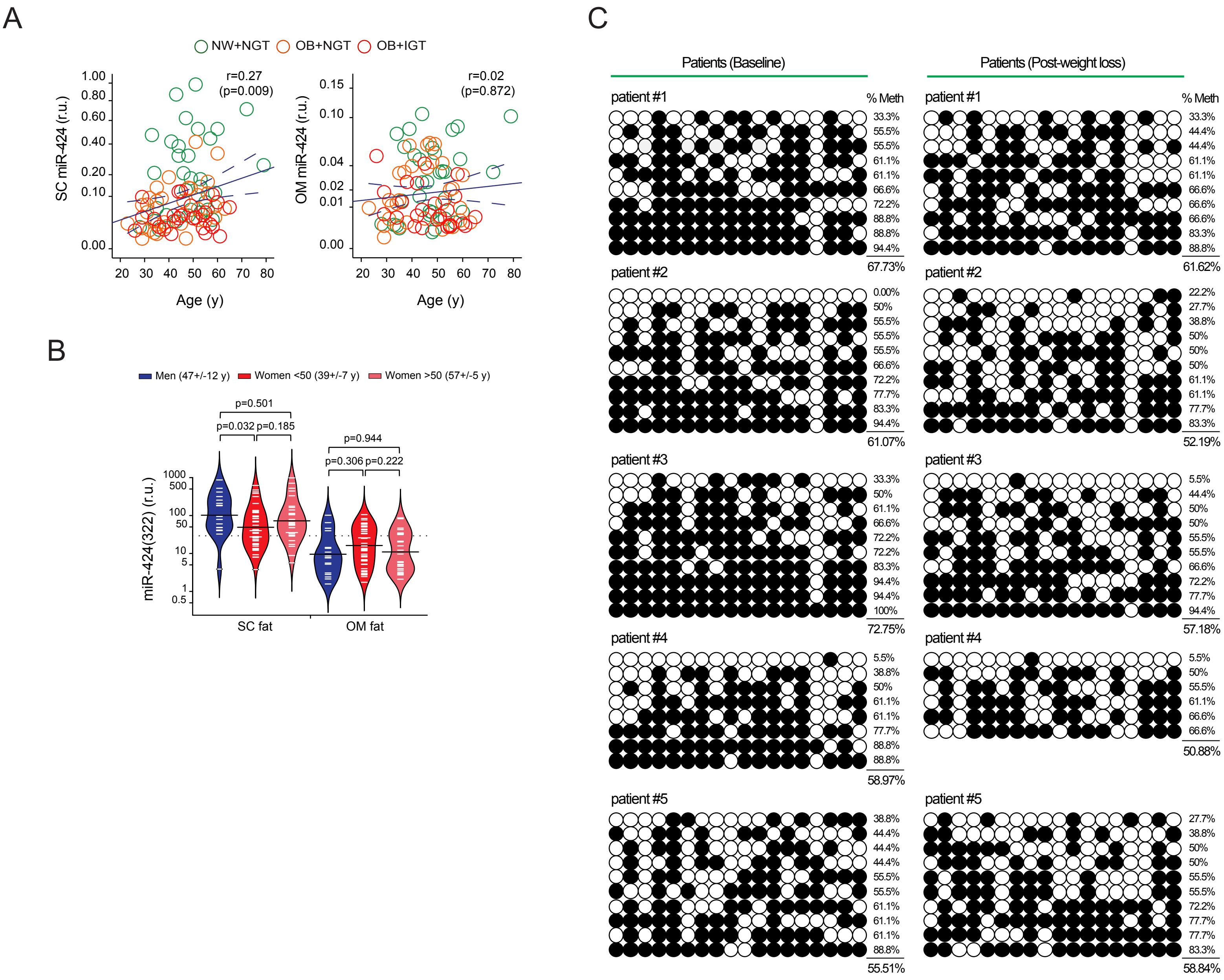

Suppl Figure-6 Rodriguez-Barrueco R

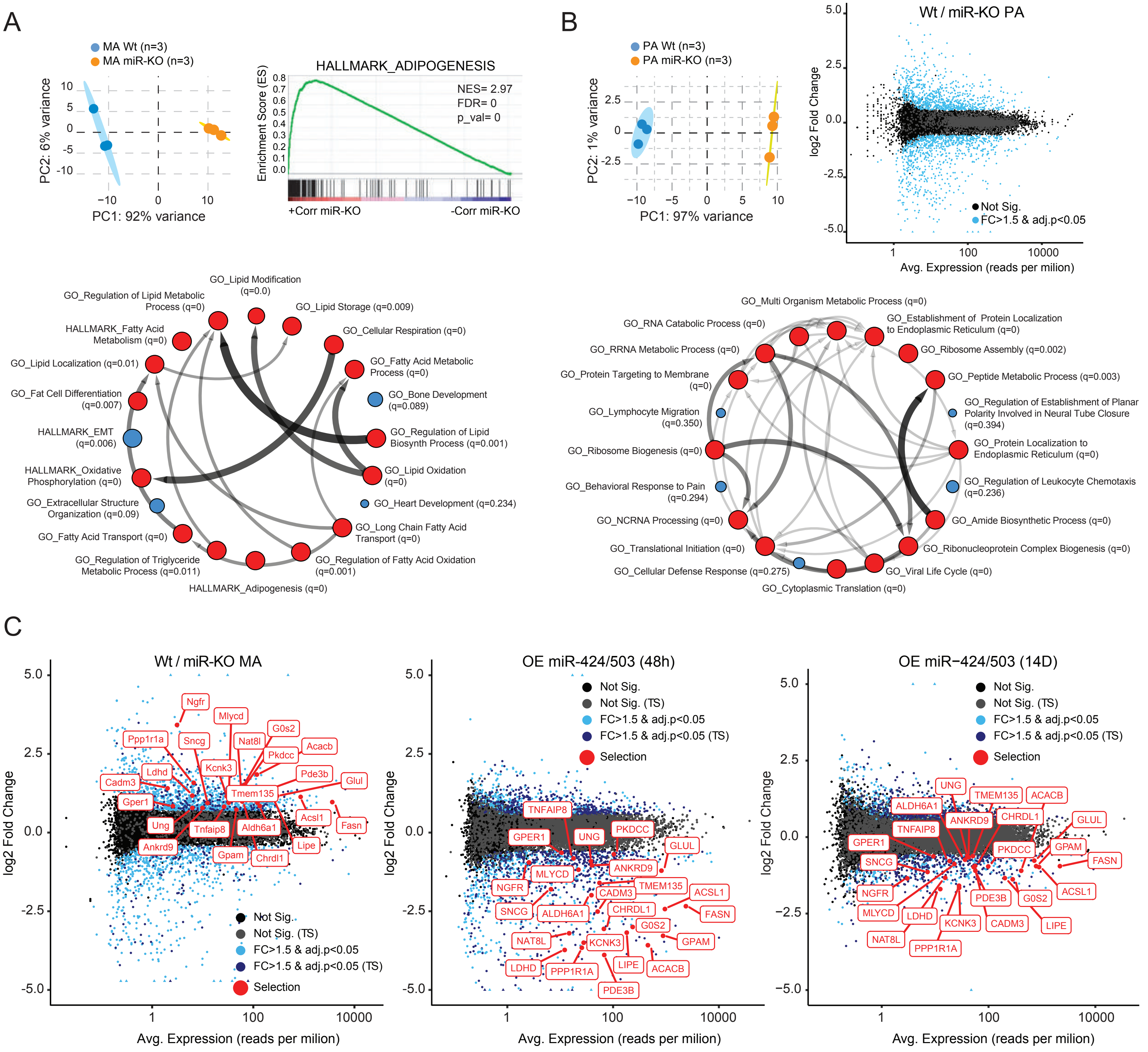

Suppl Figure-7 Rodriguez-Barrueco R

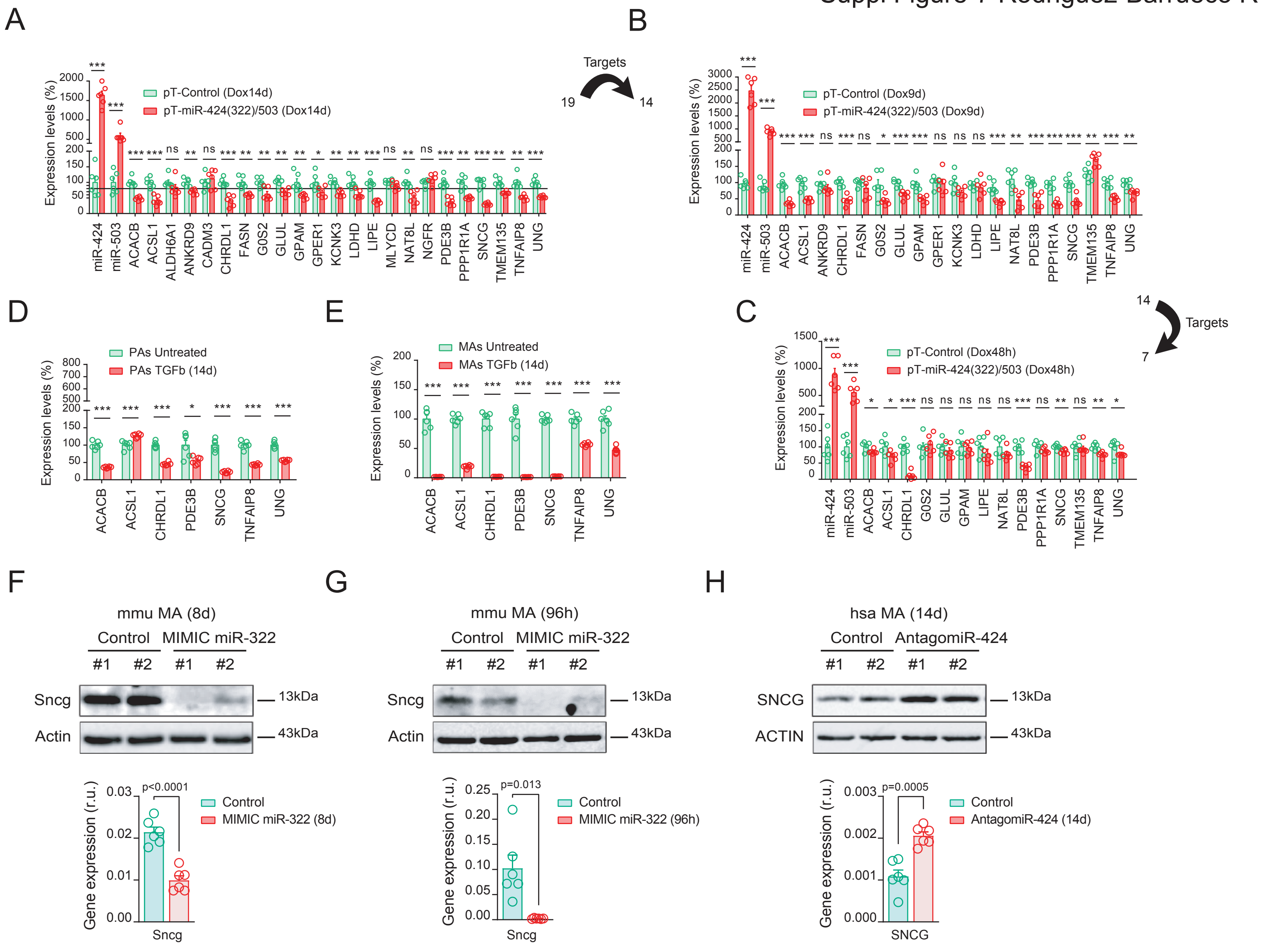

Suppl Figure-8 Rodriguez-Barrueco R

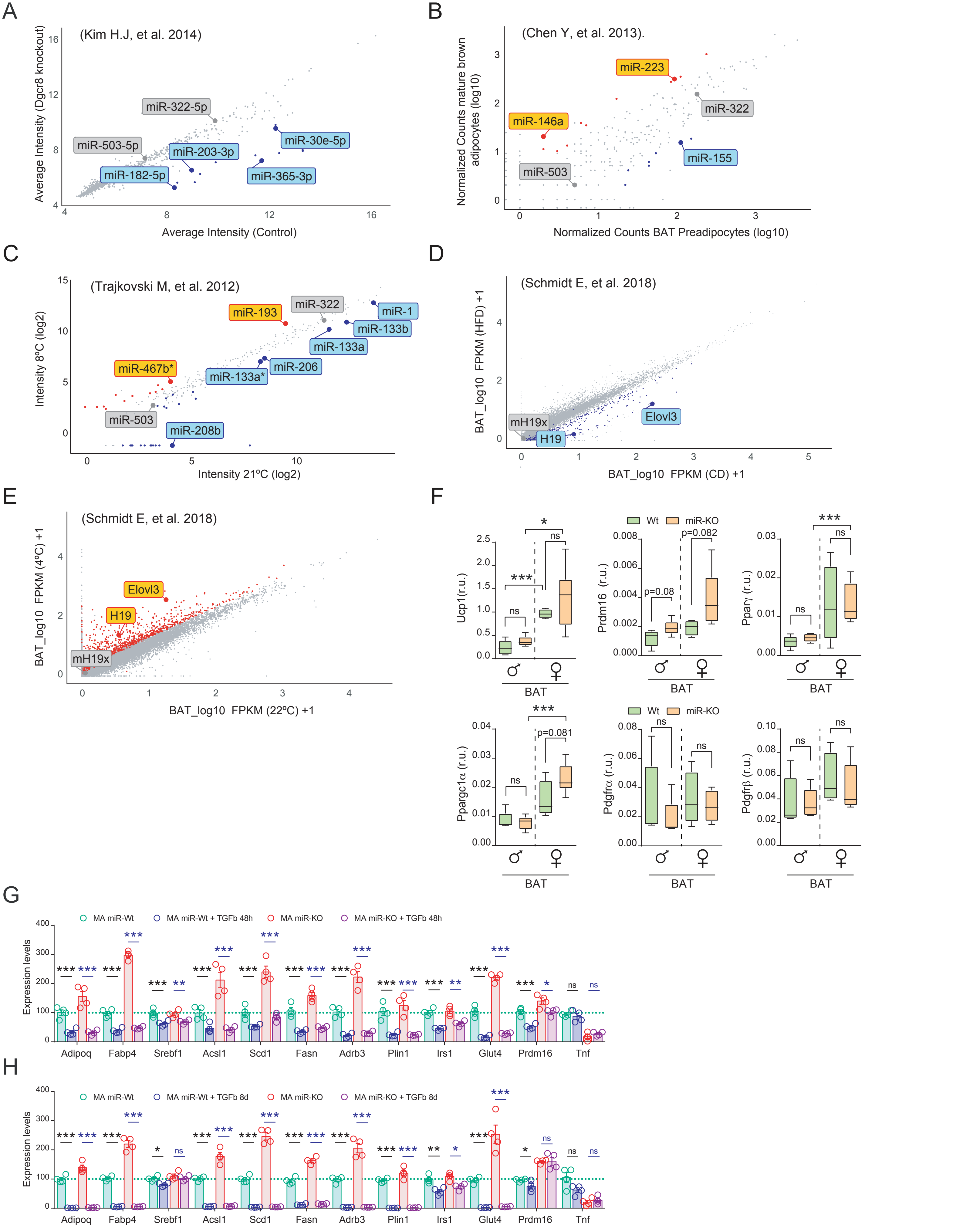

Supplement: Supplementary file 1 — Supporting Information [file ADVS-9-2104759-s001.pdf]
